# Class I HDAC overexpression promotes temozolomide resistance in glioma cells by regulating RAD18 expression

**DOI:** 10.1038/s41419-022-04751-7

**Published:** 2022-04-01

**Authors:** Daniela Hanisch, Andrea Krumm, Tamara Diehl, Carla M. Stork, Mario Dejung, Falk Butter, Ella Kim, Walburgis Brenner, Gerhard Fritz, Thomas G. Hofmann, Wynand P. Roos

**Affiliations:** 1 Institute of Toxicology, Medical Center of the University Mainz, Obere Zahlbacher Straße 67, 55131 Mainz, Germany; 2 Institute of Molecular Biology, Ackermannweg 4, 55128 Mainz, Germany; 3 Laboratory for Experimental Neurooncology, Clinic for Neurosurgery, Medical Center of the University Mainz, 55131 Mainz, Germany; 4 Department of Obstetrics and Gynecology, University Medical Center Mainz, Langenbeckstraße 1, 55131 Mainz, Germany; 5 Institute of Toxicology, Medical Faculty, Heinrich Heine University Duesseldorf, Moorenstrasse 5, 40225 Düsseldorf, Germany

**Keywords:** Acetylation, Oncogenes

## Abstract

Overexpression of histone deacetylases (HDACs) in cancer commonly causes resistance to genotoxic-based therapies. Here, we report on the novel mechanism whereby overexpressed class I HDACs increase the resistance of glioblastoma cells to the S_N_1 methylating agent temozolomide (TMZ). The chemotherapeutic TMZ triggers the activation of the DNA damage response (DDR) in resistant glioma cells, leading to DNA lesion bypass and cellular survival. Mass spectrometry analysis revealed that the catalytic activity of class I HDACs stimulates the expression of the E3 ubiquitin ligase RAD18. Furthermore, the data showed that RAD18 is part of the *O*^*6*^-methylguanine-induced DDR as TMZ induces the formation of RAD18 foci at sites of DNA damage. Downregulation of RAD18 by HDAC inhibition prevented glioma cells from activating the DDR upon TMZ exposure. Lastly, RAD18 or *O*^6^-methylguanine-DNA methyltransferase (MGMT) overexpression abolished the sensitization effect of HDAC inhibition on TMZ-exposed glioma cells. Our study describes a mechanism whereby class I HDAC overexpression in glioma cells causes resistance to TMZ treatment. HDACs accomplish this by promoting the bypass of *O*^*6*^-methylguanine DNA lesions via enhancing RAD18 expression. It also provides a treatment option with HDAC inhibition to undermine this mechanism.

## Introduction

Glioblastoma (GBM) is defined by the World Health Organization (WHO) as the most aggressive diffuse glioma of an astrocytic lineage [1]. Current therapy for patients suffering from GBM includes surgical resection, if possible, followed by radiotherapy concomitant with adjuvant chemotherapy using temozolomide (TMZ) [2]. Even with these aggressive therapeutic options, the median survival of GBM patients is only 10 months [3]. This dismal prognosis is caused by the location of the tumor and the innate cellular resistance of glioblastoma cells (hereafter glioma cells) towards the therapy employed. To date, there is only limited insight into the mechanisms underlying glioma resistance and only a few resistance markers have been identified, including *O*^6^-methylguanine-DNA methyltransferase (MGMT) [4], mutations in the isoforms 1 and 2 of isocitrate dehydrogenase (IDH1/2) [5] and mutations in mismatch repair (MMR) [6]. After initial radiotherapy and TMZ, GBM inevitably recurs. At this stage, no effective therapeutic options are currently available rendering GBM an untreatable fatal condition [7, 8]. Therefore, the need for identifying targetable resistance factors in glioma remains essential.

TMZ is an alkylating agent that methylates DNA at several sites [9], thereby interfering with the ability of glioma cells to replicate their DNA during the S-phase and triggering cell death by apoptosis [10]. The TMZ-induced DNA lesion *O*^6^-methylguanine (*O*^*6*^MeG) is repaired by MGMT [11]. In ~40% of gliomas, the expression of the *MGMT* gene is silenced due to epigenetic hypermethylation of CpG islands in its promoter region [12], leading to the loss of *MGMT* expression and increased sensitivity towards alkylating agents [4, 12]. If unrepaired, *O*^*6*^MeG mispairs with thymine during S-phase and *O*^*6*^MeG/thymine mismatches become subject to MMR [13]. MMR removes thymine, but because of the persistence of *O*^*6*^MeG in the template strand, thymine is reinserted across from *O*^*6*^MeG giving rise to futile MMR cycles [14]. Futile MMR cycles cause long stretches of single-stranded DNA to persist in the cell [15], blocking cells in the post-TMZ exposure S-phase [16] and trigger cell death [17]. These *O*^*6*^MeG-dependent replication blocking lesions activate the DNA damage response (DDR) kinases ATM and ATR [18], thereby initiating bypass in glioma cells in a tolerance mechanism that requires homologous recombination (HR) [19, 20].

Histone deacetylases (HDACs) have been shown to stimulate the activity of DNA repair [21]. HDACs are lysine deacetylases that deacetylate histones and non-histone proteins [22], contributing to multiple biological processes due to their role in the epigenetic regulation of gene expression and their influence on enzymatic activity and stability. Class I HDACs comprises HDAC1, 2, 3 and 8 and its members are often overexpressed in cancers [21]. The use of HDAC inhibitors (HDACi) as cancer therapeutics have shown promise as mono-therapeutics and in combination with other genotoxic-based therapies [23, 24]. In glioma cells and tissue, members of the class I HDACs are overexpressed [25, 26]. Furthermore, inhibition or knockdown of class I HDACs decreased the proliferation rate of glioma cells significantly [25] and sensitized the cells to lomustine and TMZ [26, 27], although the mechanism remains unclear.

Here, we report on the mechanism whereby overexpressed class I HDACs protect glioma cells against TMZ. Inhibition of class I HDACs suppresses the expression of the E3 ubiquitin-protein ligase RAD18 in glioma cells. Loss of RAD18 prevents glioma cells from initiating tolerance mechanisms for TMZ-induced DNA lesions in S-phase and consequently cell death results. These findings provide insight into an alkylating agent resistance mechanism exploited by glioma cells to resist TMZ therapy, but more importantly, provide a method for targeting this mechanism using an HDACi.

## Materials and methods

### Cells, culture, and drug treatments

The glioma cell lines LN229 (RRID:CVCL_0393), LN229 MGMT, A172 (RRID:CVCL_0131), U87MG (RRID:CVCL_0022), LN308 (RRID:CVCL_0394) and T98G (RRID:CVCL_0556) were cultivated in Dulbecco’s modified Eagle medium (DMEM) containing 10% fetal bovine serum (FBS) and penicillin/streptomycin at 37 °C in a humidified 5% CO_2_ atmosphere. LN229 and T98G were purchased from LGS Standards while A172 and U87MG were purchased from Cell Lines Service. LN308 cells were kindly provided by Prof. Weller (Laboratory of Molecular Neuro-Oncology, University Hospital and University of Zurich, Zurich, Switzerland) and were characterized [28]. Upon receipt, the cells were amplified for cryopreservation in liquid-N_2_ and freshly thawed cell stocks were used for every battery of tests. LN229 MGMT cells were generated by stable transfection with MGMT cDNA and they show strong MGMT expression and activity [20]. The glioma tumor initiating cells #1095 and their radiation-resistant clone #1095_IR were cultivated as previously described [29]. Human umbilical vein endothelial cells (HUVECs) were isolated from umbilical cords of multiple donors and cultivated as described [30, 31]. Primary human astrocytes were obtained from ScienCell and cultivated as described [32]. The primary cells were used at low passage numbers and all cells were regularly checked for mycoplasma contamination.

The handling and preparation of TMZ (provided by Dr. Geoff Margison, The University of Manchester, Centre for Occupational and Environmental Health, United Kingdom, Manchester) has been described previously [20]. MS-275 (Entinostat, Selleck Chemicals) stocks (5 mM), O^6^-benzylguanine (O^6^BG, Sigma-Aldrich) stocks (10 mM) and MERCK60 (BRD6929, Sigma-Aldrich) stocks (10 mM) were prepared by dissolving them in DMSO, aliquoting and storing them at −80 °C. The cells were treated with 1.5 µM MS-275 or MERCK60 1 h before TMZ. The cells were treated with 5 µM O^6^BG 1 h before MS-275.

### Protein extracts and western blot analysis

Western blot was performed as described [10]. The antibodies used are listed in Table S1 and S2. Proteins were visualized using the Odyssey® Infrared Imaging System (LI-COR Biotechnology) and analyzed via densitometry using ImageJ. The expression was normalized to ACTIN, HSP90 or TALIN and the untreated control was set to 1.

### Determination of apoptosis and cell cycle progression

The Sub-G_1_ assay and the Annexin V-FITC/propidium iodide double-staining methods were performed as previously described [19]. Flow cytometry was performed using the FACSCanto II (BD Biosciences) and the resulting data were analyzed with Flowing Software (Version 2.5.1) by Perttu Terho (Sub-G_1_, Annexin V/PI) or ModFit LT version 3.3 (cell cycle).

### Colony formation assay

The colony formation assay was performed as described [33]. Stained colonies containing at least 50 cells were scored.

### Neutral comet assay

DNA double-strand breaks induced by MS-275 and TMZ were detected and quantified via single-cell gel electrophoresis under neutral conditions. The cells were exposed to TMZ and MS-275 and 72 h later the comet assays were performed, as described [34].

### Transfections

LN229 cells were stably transfected with the pDRGFP for flow cytometric detection of HR-mediated DSB repair [35]. This plasmid provided the repair substrate and puromycin selector. Cells were transfected using Polyethylenimine Hydrochloride (PEI) (#24765; Polysciences) and stable clones were selected for using puromycin (0.4 µg/ml). Clones were transiently transfected with pCβASceI plasmid, as described [36] using PEI. The resulting GFP fluorescence upon HR activity was determined by flow cytometry.

LN229 and A172 cells were transiently transfected with the hRAD18-EGFP plasmid [37] using PEI. The RAD18-GFP expression was confirmed by Western blot analysis and the cell cycle distribution was analyzed 120 h after treatment using the Sub-G_1_ assay. Plasmid information is listed in Table S3.

LN229 and A172 cells were transfected with siRNA targeting RAD18 (ON-TARGETplus Human RAD18 siRNA; Dharmacon) and negative control siRNA (#AM4611; Life Technologies) using Lipofectamine RNAiMAX (Invitrogen), according to the manufacturer’s instructions. For HDAC1 and HDAC2 knockdown siHDAC1 (Dharmacon) and siHDAC2 (Santa Cruz Biotechnologies Inc.) were used.

### Mass spectrometry

Mass spectrometry was performed by separating peptides by nanoflow liquid chromatography on an EASY-nLC 1000 system (Thermo) coupled to a Q Exactive Plus mass spectrometer (Thermo). Separation, mass spectrometry settings and analysis conditions are listed in Table S4. The mass spectrometry proteomics data have been deposited to the ProteomeXchange Consortium via the PRIDE [38] partner repository with the dataset identifier PXD028683.

### Immunofluorescence staining and foci determination

A172 cells were seeded onto coverslips, exposed to treatment for 72 h and fixed using methanol:acetone (7:3). Cells were incubated with the primary antibodies, RAD18 and pATM (S1981) (Table S1) in 0.25% Triton-X100/PBS, overnight at 4 °C. The secondary antibodies (Table S2) were added for a 3 h incubation at RT in the dark. DNA was stained with TO-PRO3. Slides were mounted using Vectashield and sealed with nail polish. Micrographs were acquired by laser scanning microscopy (LSM710, Carl Zeiss MicroImaging). The foci were quantified using ImageJ.

HUVECs cells were seeded on coverslips, fixed using 4% formaldehyde and permeabilized with ice-cold methanol:acetone (1:1). The antibody staining was performed with the primary antibodies CD31 and vWF (Table S1) as previously described.

### Determination of DNA synthesis in S-phase by EdU incorporation

DNA replication was investigated by the 5-ethynyl-2´-deoxyuridine (EdU) assay, as previously described [39] except for the following changes. The cells were labeled with 10 µM EdU for 1–2 h at 37 °C, fixed with 4% formaldehyde in PBS and permeabilized with 80% EtOH incubated at –20 °C for at least 30 min. Directly after removing the EtOH by washing the cells with PBS, the Click-it reaction was performed in a total volume of 200 µl of the EdU detection mix (10 mM Sodium ascorbate, 10 mM Aminoguanidine hydrochloride, 4 mM CuSO_4_ and 44 µM 6-FAM-Azide (Lumiprobe) in PBS). The cells were incubated at RT for 60 min in the dark with gentle agitation (300 U/min). The DNA was counterstained with DAPI (1 µg/ml DAPI and RNaseA). The flow cytometric acquisition was performed using the FACSCanto II (BD Biosciences) and the results were analyzed using the Flowing Software (Version 2.5.1) and ModFit LT version 3.3 (cell cycle).

### Neurosphere assay and long-time treatment

The neurosphere assay is based on the principle of extreme limited dilution assay (ELDA) [40]. Cells were seeded in 24-well plates in a clonal density range of 0.125 to 10 cells/ml. To investigate the treatments’ influence on the self-renewal potential, the cells were treated with TMZ and MS-275 24 h after seeding. 8 weeks later the number of positive and negative wells was determined and the stem cell frequencies were calculated using the ELDA software [40].

To evaluate the influence of HDACi on cell proliferation, 50000 cells per well were seeded in a 6 well plate and treated with TMZ and MS-275 24 h later and left to grow for 24 days. Micrographs were acquired using the Cell A Imaging software (Olympus) in combination with a Zeiss Axiovert 35 microscope. For determining the size of the spheroids, the circular area was measured via ImageJ and the scale bar acquired from the micrograph.

### Quantification and statistical analysis

All data, consisting of at least three independent experiments, were evaluated using the indicated tests and expressed in graphs as mean. *p ≤ 0.05 was considered as statistically significant, **p ≤ 0.01 very significant, ***p ≤ 0.001 highly significant and ****p ≤ 0.0001 most significant. The analyses were performed using GraphPad Prism, GraphPad Software, La Jolla California USA.

## Results

### Overexpression of class I HDACs in glioblastoma contributes to temozolomide resistance

**Fig. 1:**
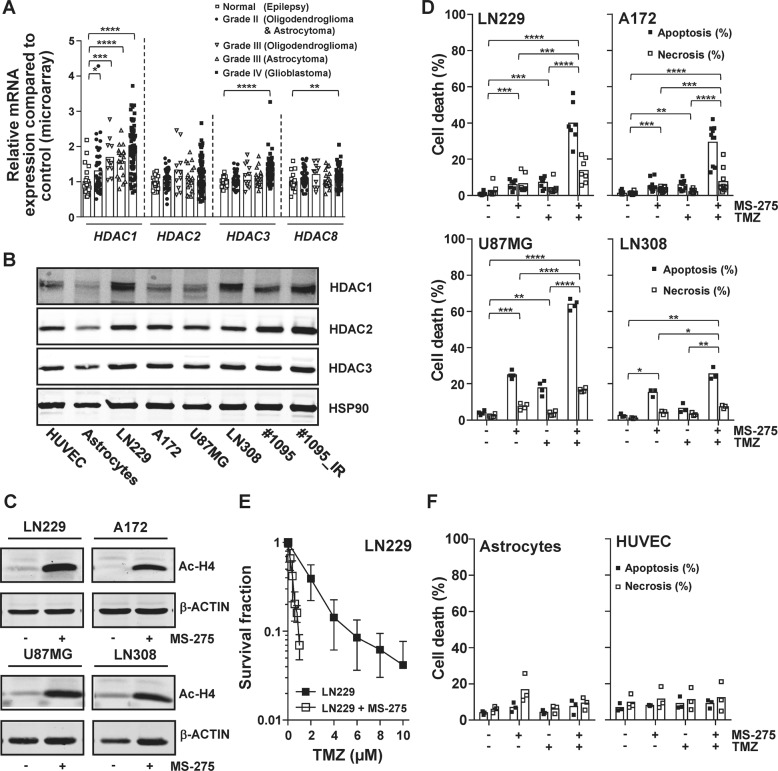
Inhibition of overexpressed HDAC1/2/3 sensitizes glioma cells towards TMZ. **A** Microarray expression data obtained from ArrayExpress of *HDAC1* (NM_004964), *HDAC2* (NM_001527), *HDAC3* (NM_003883) and *HDAC8* (NM_018486) from 23 epilepsy sufferers, 45 grade II oligodendroglioma and astrocytoma tumors, 12 grade III oligodendroglioma tumors and 81 grade IV glioblastoma tumors. Indicated significances result from a Mann–Whitney test. **B** Western blot analysis of HDAC1/2/3 protein levels in LN229, A172, U87MG, LN308 glioma cell lines and the glioma initiating cell lines #1095, #1095_IR compared to non-cancerous HUVECs and astrocytes. HSP90 served as a loading control. **C** Western blot analysis showing the influence of class I HDAC inhibition by MS-275 (1.5 µM for 72 h) on acetylated-H4 in LN229, A172, U87MG, and LN308 cells. ß-ACTIN served as a loading control. **D** Apoptosis and necrosis in the glioma cell lines LN229, A172, U87MG, and LN308 induced by the indicated drugs. Indicated significances result from a paired *t*-test. **E** Clonal survival of LN229 glioma cells exposed to TMZ in the absence and presence of MS-275 (1.5 µM). **F** Apoptosis and necrosis induced in non-cancerous cells (Astrocytes and HUVECs) exposed to the indicated drugs. Indicated significances result from a paired *t*-test. *P ≤ 0.05; **P ≤ 0.01; ***P ≤ 0.001, and ****P ≤ 0.0001. For **D** and **F** cells were exposed to MS-275 (1.5 µM), TMZ (50 µM) and TMZ/MS-275. Cell death response was determined by flow cytometric analysis of Annexin V-FITC/PI double-staining 120 h and 144 h after treatment for **D** and **F**, respectively.

As class I HDACs have been shown to play a role in cancer initiation and progression, the expression of these lysine deacetylases was examined in glioblastoma. To this end, an analysis of the publicly available dataset from ArrayExpress [41] was performed to determine the differences in *HDAC1*, *HDAC2*, *HDAC3* and *HDAC8* mRNA levels of glioblastoma compared to normal tissue (Fig. 1A). Glioblastoma significantly overexpresses the class I HDACs HDAC1/3/8. To validate whether our cellular experimental system genocopies elevated HDAC expression, the protein expression of HDAC1/2/3 were analyzed in the glioma cells LN229, A172, U87MG, LN308, #1095 and #1095_IR and compared to the expression in untransformed, primary astrocytes and HUVECs (Fig. 1B). HUVECs were authenticated by determining the expression of the HUVEC marker proteins CD31 and von Willebrand factor (vWF) [42] (Fig. S1A). The glioma cells show on average 90% higher HDAC1, 108% higher HDAC2 and 23% higher HDAC3 protein expression than astrocytes.

In order to determine the influence of class I HDACs on TMZ-resistance in glioma cells, first we had to show that HDAC inhibitors are active in these cells. Making use of the class I HDAC inhibitor MS-275 (Entinostat), which specifically inhibits HDAC1/2/3, but not HDAC8 [43], the results show that MS-275 is active in these cells as it leads to an increase in acetylated-H4, a deacetylation target of HDAC1/2 [44], in LN229, A172, U87MG and LN308 (Fig. 1C). Next, the contribution of HDAC1/2/3 to the TMZ-resistance of glioma cells was determined. LN229, A172, U87MG, and LN308 cells were exposed to low doses of TMZ (50 μM) and/or MS-275 (1.5 μM) as indicated and the cell death response was determined (Fig. 1D and Fig. S1B). While exposure to TMZ or MS-275 on their own triggered low levels of apoptosis, inhibiting HDACs in the presence of TMZ significantly sensitized all the glioma cells tested. To further substantiate our finding, clonal survival of LN229 cells upon TMZ exposure in the presence and absence of HDACi was determined (Fig. 1E). Again, MS-275 greatly sensitized LN229 cells to TMZ. In an attempt to determine glioma specificity, untransformed astrocytes and HUVECs were exposed to TMZ in the presence and absence of HDACi (Fig. 1F and Fig. S1C). Of note, MS-275 did not sensitize astrocytes nor HUVECs to TMZ-induced cell death. These data show that overexpressed class I HDACs protect glioma cells against TMZ-induced cell death.

### Inhibition of the class I HDACs 1/2/3 prevents glioma cells from activating the ATM and ATR DNA signaling axes upon TMZ treatment

**Fig. 2:**
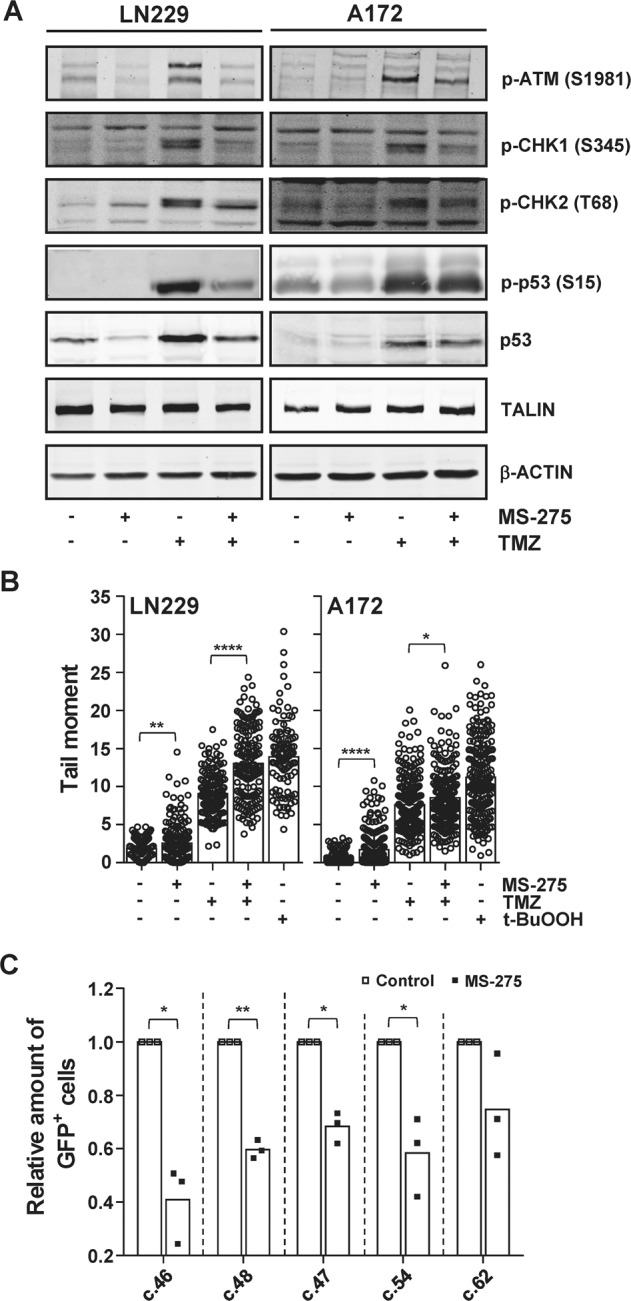
Inhibition of HDAC1/2/3 attenuates TMZ-induced DDR activation and increases TMZ-induced DSBs by impairing HR-mediated repair. **A** Effect of MS-275 on the TMZ-induced activation of the DDR kinase ATM and its targets CHK1, CHK2, p53 determined by western blot analysis. LN229 and A172 cells were exposed to indicated drugs. ß-ACTIN and TALIN served as loading controls. **B** MS-275 increases TMZ-induced DSB formation. LN229 and A172 cells were exposed to indicated drugs and DSBs were detected by neutral comet assay. Cells exposed to t-BuOOH served as a positive control. Indicated significances result from an unpaired *t*-test with Welch’s correction. **C** Effect of MS-275 on HR activity was determined in LN229 DRGFP clones c. 46, c.48, c.47, c.54, and c.62. Flow cytometric analysis was performed 96 h after transient transfection with a SceI expressing plasmid. Indicated significances result from a paired *t*-test. *P ≤ 0.05; **P ≤ 0.01; ***P ≤ 0.001 and ****P ≤ 0.0001. For **A** and **B** cells were exposed to MS-275 (1.5 µM), TMZ (50 µM) and TMZ/MS-275 and assays were performed 72 h later.

DNA double-strand breaks (DSBs) and blocked DNA replication forks activate the DDR kinases ATM, ATR, CHK1 and CHK2 [45] and these kinases protect glioma cells against TMZ-induced cell death [18]. For this reason, the influence of HDAC inhibition on TMZ-induced activation of ATM, CHK1, CHK2, and their downstream target, p53, was analyzed (Fig. 2A). ATM, CHK1, CHK2 and p53 became activated upon TMZ exposure as the levels of pATM (S1981), the site that is autophosphorylated upon ATM activation [46], p-CHK1 (S345), the site phosphorylated by ATR upon its activation [47], and p-CHK2 (T68), the site phosphorylated by ATM upon its activation [48], increased following TMZ exposure. HDACi caused a decrease in TMZ-induced ATM, CHK1 and CHK2 activation. This finding warranted further investigation to determine whether HDACi decreases the amount of TMZ-induced DNA damage.

The clinically relevant DNA lesion induced by TMZ, *O*^6^MeG, is processed by MMR into DSBs in a replication-dependent manner [49–51]. For this reason, the formation of DSBs upon TMZ and/or MS-275 was analyzed using the neutral comet (Fig. 2B and S2A) assay. TMZ and MS-275 induced DSBs in both LN229 and A172 cells. Combining TMZ with the HDACi increased the amount of DSBs in LN229 and A172 cells. These small increases in DSB formation upon TMZ and MS-275 exposure might reflect the insensitivity of the neutral comet assay in detecting one-ended DSBs formed at collapsed replication forks, therefore we addressed the possible influence of HDACi on bypass of stalled replication forks by making use of an HR activity assay [35] (Fig. 2C and Fig. S2B). Four out of five HR reporter LN229DRGFP clones showed a significant decrease in HR-mediated DSB repair upon MS-275 exposure. These results confirm the previous observation that MS-275 impairs HR-mediated DSB repair, leading to an increase in unrepaired TMZ-induced DSBs. The neutral comet results and the HR reporter assay point to a possible DNA repair defect induced by HDACi, which would explain the increase in cell death but does not address the decrease in DDR kinase activation. As the activation of these kinases can protect glioma cells from TMZ-induced cell death [18], we hypothesized that the loss of DDR kinase activation, or a premature timing of their activation, may be the reason underlying the sensitization of glioma cells to TMZ upon HDAC inhibition.

### The influence of HDAC1/2/3 on TMZ-induced protein expression in glioma cells

**Fig. 3:**
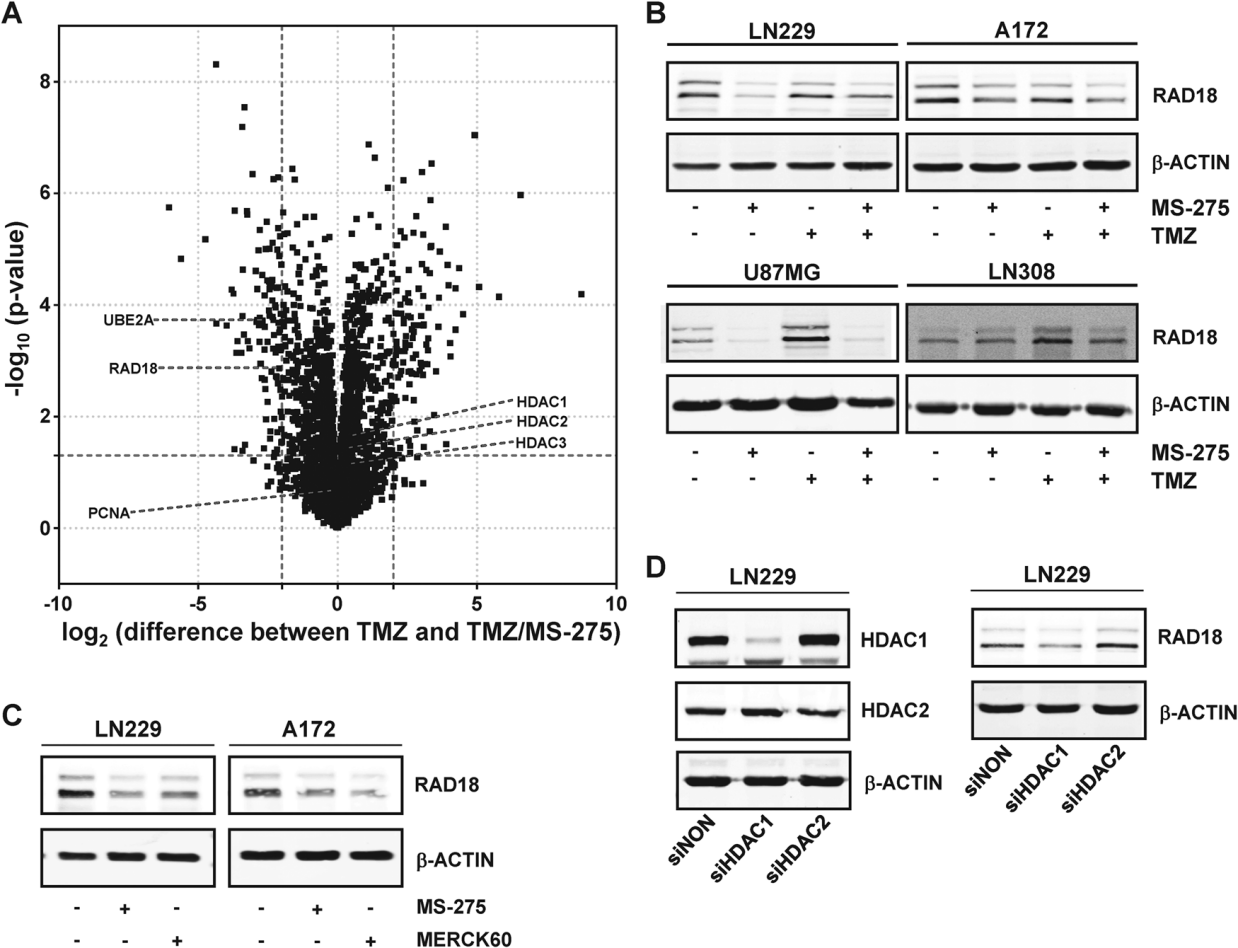
Influence of HDACi on TMZ-induced protein expression. **A** Volcano Plot of data obtained by quantitative mass spectrometry comparing LN229 cells exposed to TMZ and TMZ/MS-275 for 72 h. Approximately 4000 proteins were detected in quadruple samples, of which 196 were significantly altered by MS-275. **B** Influence of MS-275 on the expression of RAD18 in glioma cells. The RAD18 protein expression of glioma cells (LN229, A172, U87MG, LN308) upon treatment was determined by Western blotting. Cells were exposed to MS-275 (1.5 µM), TMZ (50 µM) and TMZ/MS-275 and the assay was performed 72 h later. **C** Influence of MERCK60 on the expression of RAD18 in LN229 and A172 cells as determined by western blotting. Cells were exposed to MS-275 (1.5 µM) or MERCK60 (1.5 µM) and the assay was performed 72 h later. **D** Influence of HDAC1 or HDAC2 on the expression of RAD18 in LN229 cells as determined by western blotting. HDAC1 and HDAC2 were knocked down using RNAi and the assay was performed 144 h later. For **B**, **C**, and **D** ß-ACTIN served as loading control.

To identify how HDACi alters protein expression that could account for the decreased DDR kinase activation and consequential decreased TMZ-induced lesion bypass, protein expression levels were determined by mass spectrometry. The mass spectrometry data (Fig. 3A) showed that HDACi causes the downregulation of RAD18 and UBE2A, an E3 ubiquitin ligase and an E2 ubiquitin-conjugating enzyme respectively, in TMZ-exposed glioma cells. RAD18, together with UBE2A, initiate lesion bypass at stalled replication forks by mono-ubiquitinating PCNA at K164 thereby protecting cells from DNA damage-induced replication stress [52–54]. To determine whether the downregulation of RAD18 by HDACi is a general occurrence in glioma cells the influence of HDAC inhibition on RAD18 protein level was determined in a panel of glioma cells. MS-275 caused the downregulation of RAD18 in all TMZ-exposed glioma cell lines tested (Fig. 3B). To determine which HDAC is responsible for the regulation of RAD18, cells were exposed to MERCK60, a specific brain-penetrant inhibitor of HDAC1 and HDAC2 [55] and knocked down HDAC1 and HDAC2 by siRNA. Inhibition of HDAC1 and HDAC2 by MERCK60 downregulated RAD18 (Fig. 3C). As only the knockdown of HDAC1 decreased the RAD18 protein level (Fig. 3D) we conclude that the inhibition of HDAC1 causes the downregulation of RAD18. As TMZ induces DNA replication blocking lesions, the hypothetical role of RAD18 in the TMZ-induced DNA damage response was investigated.

### RAD18 protects glioma cells against TMZ-induced cell death

**Fig. 4:**
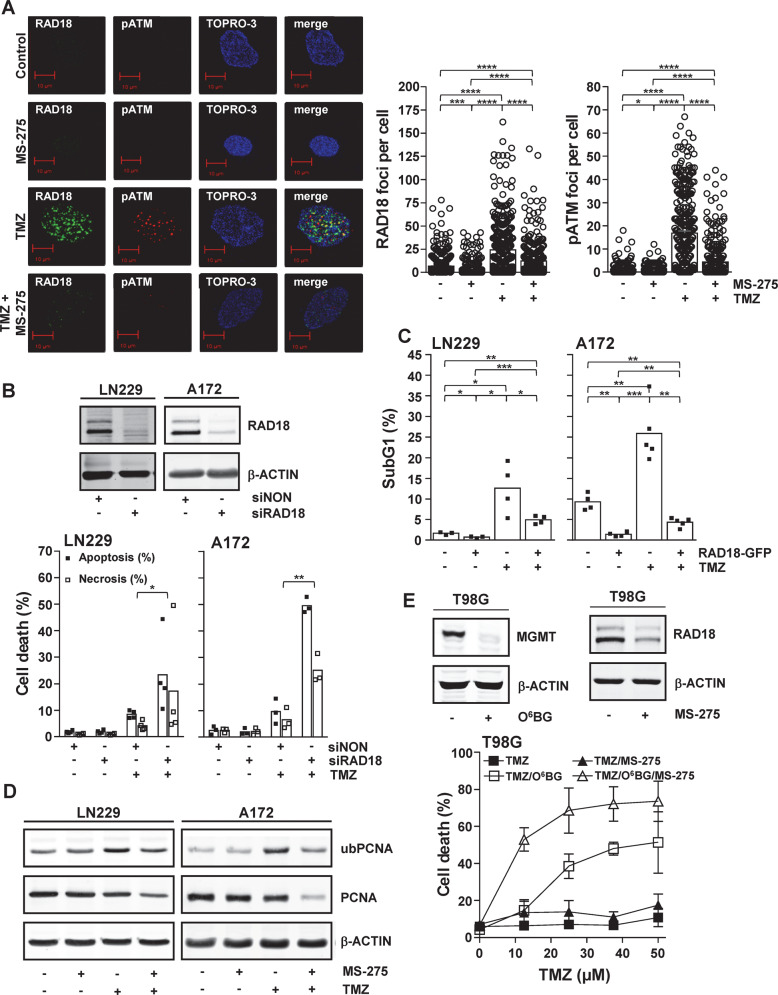
Role of RAD18 in the TMZ-induced DDR. **A** TMZ induces pATM (S1981) and RAD18 foci. A172 cells were exposed to indicated drugs and RAD18 and pATM (S1981) foci were quantified following immunofluorescent staining. For example left panel, for quantification right panel. Nuclei were stained with TO-PRO-3 (magnification, x630, scale bar, 10 µm). Indicated significances result from an unpaired t-test with Welch’s correction. **B** siRNA-mediated knockdown of RAD18 sensitizes glioma cells towards TMZ exposure. Following RAD18 knockdown, assessed by western blot analysis (Top), cell death response was determined after TMZ exposure in LN229 and A172 cells (Bottom). As control, cells were transfected with a non-targeting siRNA (siNON). Indicated significances result from a ratio paired *t*-test. **C** Overexpressed RAD18 protects glioma cells from entering TMZ-induced cell death. LN229 and A172 cells were exposed to TMZ and transiently transfected with a RAD18-EGFP expressing plasmid. **D** HDACi prevents TMZ-induced mono-ubiquitination of PCNA as determined by western blot analysis. Glioma cells (LN229, A172) were exposed to indicated drugs. ß-ACTIN served as a loading control. **E** HDACi sensitizes glioma cells towards the *O*^6^MeG lesion induced by TMZ. In MGMT expressing T98G cells (insert, left), MS-275 (1.5 µM) downregulates RAD18 (insert, right) and sensitizes toward TMZ once MGMT is depleted by O^6^BG (5 µM) (bottom). Cell death represents the sum of apoptosis and necroses. Indicated significances result from an unpaired *t*-test. *P ≤ 0.05; **P ≤ 0.01; ***P ≤ 0.001 and ****P ≤ 0.0001. For **A** and **D** cells were exposed to MS-275 (1.5 µM), TMZ (50 µM) and TMZ/MS-275 and assays were performed 72 h later and 120 h later for **E**. For **B** and **E** the cell death response was determined by flow cytometric analysis of Annexin V-FITC/PI double-stained cells and for **C** by Sub-G1 analysis 120 h after the exposure to TMZ (50 µM).

RAD18 and UBE2A initiate the bypass of replication blocking lesions induced by various DNA-damaging agents [52, 56, 57], thus protecting cells against DNA damage-induced replication stress. Furthermore, when blocking DNA replication using aphidicolin it has been shown that RAD18 is required for the activation of ATM [58]. To further elucidate the role of RAD18 in the TMZ-induced DDR, RAD18 and pATM (S1981) foci formation was examined by immunofluorescent staining in glioma cells exposed to MS-275, TMZ and TMZ/MS-275 (Fig. 4A). TMZ induced the formation of RAD18 and pATM foci. Similar to what was observed in the Western blot results, MS-275 decreases the amount of RAD18 and pATM foci induced by TMZ. These data support the hypothetical role of RAD18 in the TMZ-induced DDR.

To assign a causal role for the loss of RAD18 upon HDAC inhibition in the sensitization of glioma cells towards TMZ-induced cell death, RAD18 was knocked down using siRNA (Fig. 4B, upper panel). Knockdown of RAD18 did not influence the basal cell death level in LN229 and A172 cells compared to the non-targeting siRNA (siNON) control (Fig. 4B, lower panel). However, it caused a significant increase in TMZ-induced cell death in both LN229 and A172 glioma cell lines, demonstrating that RAD18 knockdown phenocopies the sensitization caused by HDACi in combination with TMZ exposure. Having shown that downregulation of RAD18 sensitizes glioma cells against TMZ-induced cell death, next we analyzed whether overexpression of RAD18 would protect these cells. Overexpressing RAD18-GFP in LN229 and A172 cells significantly protected these cells against TMZ-induced cell death (Fig. 4C and Fig. S3A). Collectively, our results show that RAD18 mediates TMZ-resistance of glioma cells and its depletion reduces cancer cell resistance.

### RAD18 protects glioma cells from TMZ-induced *O*^6^MeG lesions

RAD18 mono-ubiquitinates PCNA to initiate bypass of replication blocking lesions [52, 56, 57]. To unravel the role of RAD18 in the TMZ-induced DDR, the mono-ubiquitination of PCNA upon TMZ-induced replication stress was determined. To this end, LN229 and A172 cells were exposed to TMZ and samples were collected at various time points after TMZ exposure (2–72 h) (Fig. S3B). Both cell lines showed a slight increase in ub-PCNA at early time points after TMZ exposure (2–12 h). However, at later time points (16–72 h), the level of ub-PCNA constantly increased. These results confirm that the processing of *O*^6^MeG lesion by MMR during the subsequent S-phases causes ub-PCNA. To determine whether the decrease in RAD18 expression caused by HDAC inhibition would influence the TMZ-induced mono-ubiquitination of PCNA, LN229, A172, and U87MG cells were exposed to MS-275, TMZ, and TMZ/MS-275 and the level of ub-PCNA was determined (Fig. 4D and S3C). In all cell lines, MS-275 on its own did not influence the basal level of ub-PCNA, TMZ increased the level of ub-PCNA while MS-275 attenuated the TMZ-induced ub-PCNA level. We should state that MS-275 in combination with TMZ lead to a decrease in total PCNA levels, which might reflect the influence that inhibition of HDACs have on the stability of PCNA as acetylated PCNA becomes degraded following DNA damage [59].

To support the hypothesis that the replication-dependent processing of the TMZ-induced *O*^6^MeG lesion by MMR causes replication blocking lesions, which require RAD18 for cellular survival, TMZ-induced cell death in T98G cells that express MGMT and LN229 cells stably reconstituted for MGMT expression was investigated (Figs. 4E and S3D). As stated, MGMT repairs *O*^6^MeG lesions and if sensitization to TMZ by HDAC inhibition is lost in MGMT expressing cells we could conclude that MS-275 sensitizes to this lesion. To that end, T98G cells were exposed to increasing concentrations of TMZ in the presence or absence of the MGMT inhibitor *O*^6^BG and/or in the presence or absence of MS-275. *O*^6^BG was able to deplete MGMT in T98G cells (Fig. 4E, upper left insert) and MS-275 was able to downregulate RAD18 (Fig. 4E, upper right insert). In the presence of MGMT, T98G cells were resistant to TMZ and upon HDACi with MS-275 only a slight non-significant increase in cell death was observed (Fig. 4E, lower panel). Upon inhibition of MGMT with *O*^6^BG, TMZ induced cell death in a dose-dependent manner and upon simultaneous inhibition of MGMT and HDACs a further, and significant, increase in cell death was observed (Fig. 4E, lower panel). LN229 MGMT expressing cells were exposed to MS-275, TMZ and TMZ/MS-275 and the cell death response was determined (Fig. S3D, right panel). MS-275 increased cell death slightly, while TMZ in LN229 MGMT expressing cells did not induce cell death to a similar level observed in the parental cells (Fig. 1D). Furthermore, the sensitization effect of MS-275 on TMZ-induced cell death was lost in the presence of MGMT. Collectively, the data obtained in T98G and LN229MGMT cells demonstrated that HDAC inhibition by MS-275 sensitizes glioma cells to TMZ-induced *O*^6^MeG lesions.

### Downregulation of RAD18 by HDACi impairs DNA synthesis and enhances TMZ-induced S-phase accumulation

**Fig. 5:**
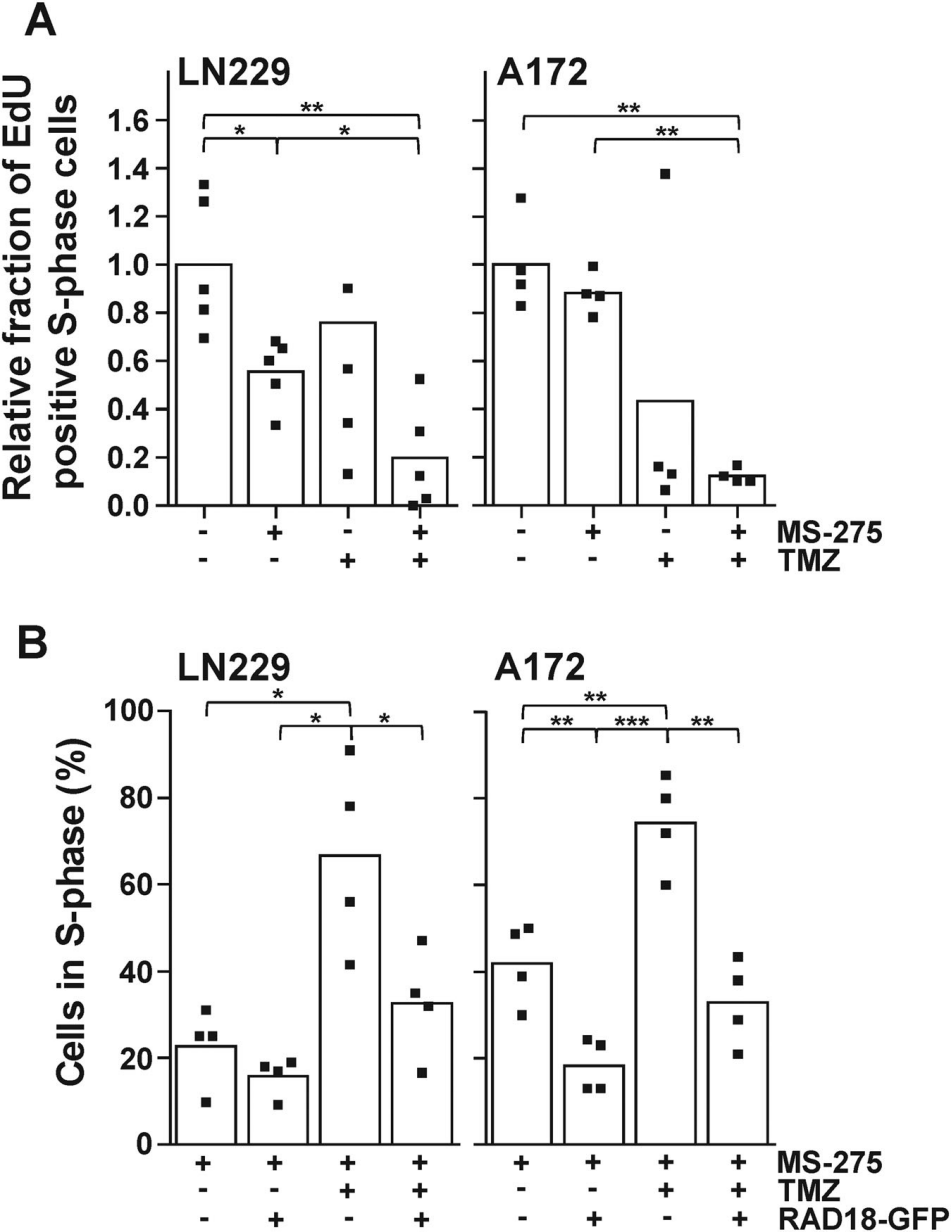
Effect of RAD18 downregulation by MS-275 on DNA replication and cell cycle progression. **A** Quantification of EdU incorporation in LN229 and A172 cells exposed to indicated drugs. EdU incorporation and cell cycle distribution were determined by flow cytometric analysis. **B** Overexpressed RAD18 prevents TMZ-induced S-phase accumulation independent of MS-275. Cells were transiently transfected with RAD18-EGFP plasmid and cell cycle distribution was determined by Sub-G1 analysis 120 h after drug exposure. *P* ≤ 0.05; **P ≤ 0.01 and ***P ≤ 0.001. Indicated significances result from an unpaired *t*-test with Welch’s correction. For **A** and **B** cells were exposed to MS-275 (1.5 µM), TMZ (50 µM) and TMZ/MS-275 and assays were performed 72 h and 120 h later, respectively.

To test whether downregulation of RAD18 by HDACi leads to a decrease in DNA synthesis because of impaired TMZ-induced lesion bypass, LN229 and A172 cells were exposed to MS-275, TMZ and TMZ/MS-275 and DNA synthesis was monitored by analyzing the incorporation rate of the thymidine analog EdU (Fig. 5A and Fig. S4A). The downregulation of RAD18 by MS-275 caused a decrease in EdU positivity in LN229 and A172 cells. TMZ-induced lesions also caused a decrease in EdU-positive cells. The combination of TMZ-induced DNA lesions and impaired RAD18-dependent bypass initiation by MS-275 further decreased EdU incorporation. To determine whether RAD18 plays a causative role in overcoming the TMZ/MS-275-induced S-phase accumulation, RAD18-GFP was transiently overexpressed in LN229 and A172 cells and the cell cycle distribution was determined after exposure to MS-275 and TMZ/MS-275 (Fig. 5B and Fig. S4B). The untransfected, GFP-negative cell population showed an accumulation of cells in S-phase upon TMZ/MS-275 exposure, while the RAD18-GFP expressing cells showed a significant decrease in TMZ/MS-275-induced S-phase accumulation. Having been able to rescue glioma cells from the TMZ/MS-275 induced S-phase accumulation by overexpressing RAD18-GFP, we conclude that RAD18 is required for bypassing TMZ-induced *O*^6^MeG MMR intermediates in S-phase.

### HDACi affects proliferation and self-renewal of patient-derived glioma initiating cells

**Fig. 6:**
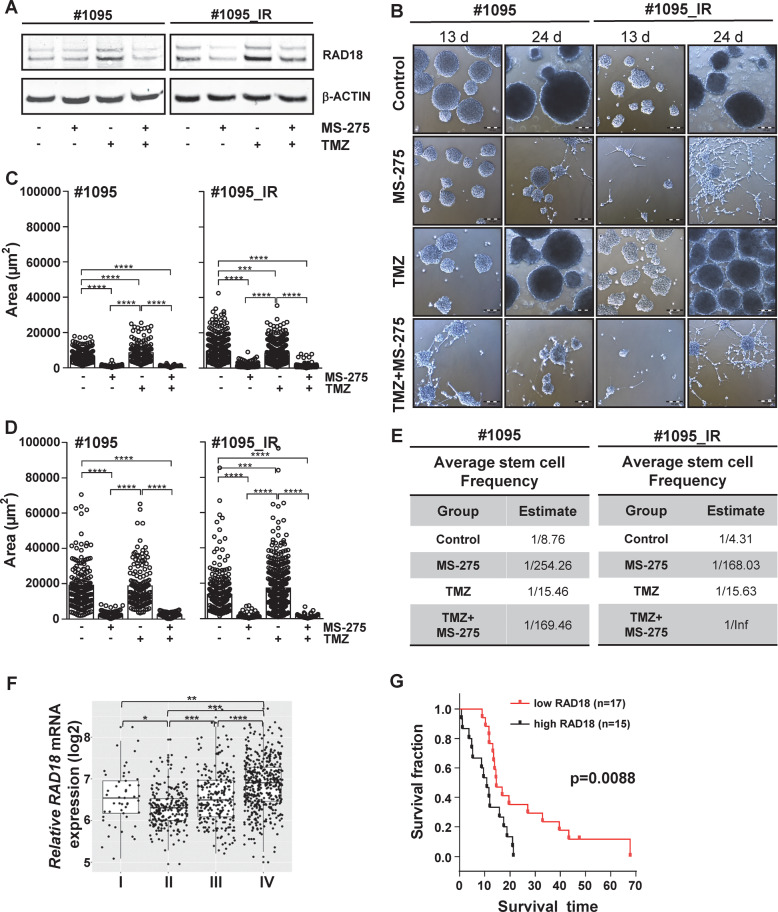
Effect of MS-275 on glioma initiating cells and the role of RAD18 in glioma patients. **A** Downregulation of RAD18 protein expression upon MS-275 exposure. Glioma initiating cells #1095 and #1095_IR were exposed to indicated drugs for 72 h. ß-ACTIN served as a loading control. **B** HDACi prevents the proliferation of glioma initiating cells. Micrographs of #1095 and #1095_IR cells after 13 and 24 days of indicated drug exposure (magnification, x40, scale bar, 100 µm). **C** Quantification of #1095 and #1095_IR spheroid size 13 days after treatment. **D** Quantification of #1095 and #1095_IR spheroid size 24 days after treatment. **E** MS-275 impairs the self-renewal potential of glioma initiating cells. Comparison of self-renewal potential in #1095 and #1095_IR upon indicated drug exposure by ELDA. The average stem cell frequency of both cell lines under indicated treatments result from three independent experiments and are shown. **F** *RAD18* mRNA expression in glioma grade I, II, III, IV. Log2 fold-changes in expression are represented as box and dot-plots of each subtype and the indicated significances *P ≤ 0.05; **P ≤ 0.01 and ***P ≤ 0.001 result from *t*-test. **G** *RAD18* mRNA expression correlates with survival of glioblastoma patients. Kaplan–Meier plot comparing the survival of GBM patients suffering from high RAD18-expressing glioblastomas (*n* = 15) and low *RAD18*-expressing glioblastomas (*n* = 17). Indicated significance result from log-rank test. For **A**, **B**, **C**, and **D** cells were exposed to MS-275 (1.5 µM), TMZ (50 µM) and TMZ/MS-275. Inf = infinity. *P* ≤ 0.001 and ****P ≤ 0.0001. Indicated significances result from an unpaired *t*-test.

As HDACi sensitizes different glioma cell lines by attenuating RAD18 expression (Fig. 1D), the question arose whether patient-derived glioma initiating cells would also be affected by MS-275 exposure. To this end, two MGMT-negative patient-derived glioma initiating cell lines, #1095 and its radiation-resistant counterpart #1095_IR (Fig. S5A–D), were exposed to MS-275, TMZ and TMZ/MS-275 and the RAD18 protein expression was determined (Fig. 6A). Consistent with our previous results, MS-275 decreased the RAD18 protein expression in #1095 and #1095_IR and prevented TMZ from inducing an increase of RAD18. During these experiments, a severe change in morphology and proliferation was observed upon HDACi in both cell lines. To address the effect of HDACi on proliferation and differentiation, cells were exposed to MS-275, TMZ and TMZ/MS-275 and monitored for 24 days. The resulting micrographs (Fig. 6B) illustrate that one-time exposure to MS-275 severely decreased cell proliferation in both cell lines (Fig. 6C, D). TMZ decreased proliferation initially in #1095_IR (day 13) (Fig. 6C) but the cells recovered (day 24) (Fig. 6D). In the TMZ/MS-275 treated cells, proliferation was prevented in both cell lines (Fig. 6C, D).

Self-renewal propensity is the key biological property of glioma initiating cells distinguishing them from non-initiating glioma cells. Making use of the ELDA, the influence of MS-275, TMZ and TMZ/MS-275 on the self-renewal potential of these two cell lines was investigated (Fig. 6E and Fig. S5E, F). Upon HDACi, the stem cell frequency in #1095 decreased (1/254.26) compared to control (1/8.76), while TMZ only had a small effect on the self-renewal potential (1/15.46). The combination of HDACi and TMZ decreased the stem cell frequency to 1/169.46, compared to TMZ on its own. The results for the radiation-resistant cell line #1095_IR were similar; control (1/4.31), MS-275 (1/168.03); TMZ (1/15.63), except that upon TMZ/MS-725 exposure the self-renewal potential was completely abolished (1/Inf). Multiple comparisons revealed that the differences in stem cell frequency are significant, except when comparing #1095 cells exposed to MS-275 and TMZ/MS-275 (Fig. S5F). Interestingly, the combination of HDACi and TMZ exerted different effects on the self-renewal propensity of non-radiated or radiation-selected patient-derived glioma initiating cells (#1095 and #1095_IR, respectively). In non-radiated patient-derived glioma initiating cells, the reduction of self-renewal potential caused by MS-275 alone was not reduced further in the presence of TMZ. However, the combined treatment with MS-275 and TMZ led to a drastic impairment of self-renewal (Fig. 6E) in #1095_IR cells representing radiation-resistant populations of patient-derived glioma initiating cells.

### RAD18 expression correlates with glioma patient survival

To demonstrate the role of RAD18 in TMZ-resistance in patients suffering from glioma, the subtype-specific *RAD18* mRNA expression pattern of glioma samples grade I-IV was investigated using the Gene Expression database of Normal and Tumor tissues 2 (GENT2) [60] on multiple microarray datasets (Table S5 and Fig. 6F). Similar to what was observed for *HDAC1* expression (Fig. 1A), *RAD18* is also overexpressed in grade IV tumors (Fig. 6F). Comparing the overall survival of patients suffering from glioblastoma with high *RAD18* expression to the ones with low *RAD18* expression showed that the group with high levels had a significantly decreased overall survival compared to the low expressors (Fig. 6G). Therefore, *RAD18* expression correlates with the survival of glioblastoma patients and this supports the hypothesis that RAD18 protects glioblastoma cells against the current therapy options.

## Discussion

Although the processing of DNA lesions induced by the alkylating agent TMZ has been studied extensively, the survival rate of glioblastoma patients remains low due to the high recurrence rate. As treatment options for recurrent glioblastomas remain scarce, we address the role of HDAC1/2/3 in TMZ-resistance. First, we demonstrated that HDAC1/3/8 are overexpressed in glioblastoma tissue compared to normal brain tissue and that HDAC1/2/3 are overexpressed in glioma and glioma initiating cell lines. For this study we made use of the class I HDAC inhibitor MS-275 as in vivo studies in rats have demonstrated that MS-275 can pass the blood-brain barrier leading to an increased level of acetylated-H3 in brain tissue [61]. Furthermore, injecting MS-275 intratumorally once, 7 days after orthotopic implantation of glioma cells in rats, drastically reduced the tumor volume, while the neurotoxic potential of MS-275, which was evaluated using ex vivo experiments, remained low [61]. Here, we showed that HDAC inhibition by MS-275 sensitizes glioma cells towards TMZ while leaving non-cancerous cells unaffected as HUVECs and primary human astrocytes were not sensitized towards TMZ. This is in agreement with a previous study that reported that MS-275 enhances the pro-apoptotic properties of TMZ in glioma cells [27]. Here, we demonstrated that the TMZ-induced DNA lesion *O*^6^MeG is causative for this sensitization and further elucidated the mechanism whereby glioma cells are sensitized.

Inhibiting class I HDACs upon TMZ exposure led to an increase in unrepaired TMZ-induced DSBs and, surprisingly, a decrease in TMZ-induced DDR kinase activation. Making use of mass spectrometry we found that MS-275 causes a decrease in the protein expression of the E3 ubiquitin ligase RAD18 in glioma cells. RAD18 is known to protect cells against different DNA lesions [52–54] by promoting the mono-ubiquitination of PCNA and thereby initiating their bypass during DNA replication. Of interest, the *RAD18* expression level in gliomas is an indicator for the poorer overall survival of patients suffering from this disease. In this study, it was shown that RAD18 protects glioma cells from entering TMZ-induced cell death, as the siRNA-mediated knockdown of RAD18 sensitized glioma cells towards TMZ treatment and exogenous overexpression of RAD18 prevented TMZ-induced cell death.

RAD18, along with the E2 ubiquitin conjugase UBE2A, which was also downregulated upon HDAC inhibition, mono-ubiquitinates PCNA to initiate the tolerance mechanism of DNA replication blocking lesion bypass by either switching the replication polymerase to a translesion polymerase [62] or upon further ubiquitination of PCNA to an HR-dependent template switching mechanism [63]. TMZ-induced *O*^6^MeG lesions block DNA synthesis in the post-exposure S-phase due to their processing by MMR. These DNA structures are bypassed in an HR-dependent manner [20, 64]. TMZ-induced the mono-ubiquitination of PCNA, indicating that the DNA lesions (DNA structures) formed during the processing of *O*^6^MeG by MMR require RAD18-mediated bypass. This bypass, however, attenuated DNA replication, as determined by EdU incorporation, leading to an accumulation of cells in the S-phase. Inhibition of HDACs in TMZ-exposed cells decreased RAD18 expression, decreased the mono-ubiquitination of PCNA and decreased the DNA replication rate even further causing more cells to accumulate in S-phase.

The MS-275-mediated downregulation of RAD18 prevented not only the increase in ub-PCNA but also increased TMZ-induced DSB formation by impairing HR-mediated repair. RAD18 is known to be essential for the functional interaction between FANCD2, RAD51 and BRCA2, promoting HR [65], which could account for the observed decrease in HR activity. Of note, the decrease in DSB repair did not lead to increased activation of the DDR kinase ATM and downstream targets, there was instead a decrease in TMZ-induced DDR kinase activation upon HDAC inhibition. It has been shown that the mono-ubiquitination of PCNA by RAD18 mediates the activation of the DDR kinase ATM by promoting the crosstalk between the modified PCNA, WRN-interacting protein 1 (WRNIP1) and ATM interactor (ATMIN), leading to the activation of ATM [58]. In our experimental setup, we observed that TMZ induced the formation of pATM (S1981) and RAD18 foci, while HDAC inhibition prevented the formation of these foci. This shows that RAD18 plays an essential role in the TMZ-induced DNA damage response.

How class I HDACs regulate the expression of RAD18 remains an open question. In human osteosarcoma cell lines, it was shown that RAD18 is a transcriptional target for the transcription factor E2F3 [66]. Additionally, the DNA damage-induced (Cisplatin) mono-ubiquitination of PCNA was lost upon E2F3 knockdown but could be rescued by exogenous RAD18 expression. Whether HDACs regulate the expression of RAD18 through E2F3 in gliomas is still unknown.

Having shown that HDACi sensitizes glioma cells towards TMZ treatment, we also investigated whether this treatment schedule is applicable for patient-derived glioma initiating cells. These cells are proposed to be one of the reasons for the high recurrence rate in glioblastoma patients as they have a very high self-renewal capacity and they can recapitulate the tumor of origin within months after therapy. Here we could show that MS-275 on its own drastically reduces the self-renewal potential and cell proliferation of glioma initiating cells. Additionally, these cells underwent a drastic change in morphology upon MS-275 treatment. In support of our findings, inhibition of HDAC3 by RGFP966 was shown to promote the differentiation of glioma initiating cells by impairing the TGF-ß signaling pathway mediated by SMAD7 [67].

In conclusion, glioma cells require RAD18 for the bypass of the TMZ-induced DNA lesion *O*^6^MeG by a mechanism that involves the ubiquitination of PCNA and the activation of the TMZ-induced DDR. Class I HDACs stimulate the expression of RAD18, and consequently, their inhibition leads to decreased RAD18, decreased DNA lesion bypass, decreased ubiquitinated PCNA and increased cell death. Our study identifies a novel mechanism whereby HDACs promote glioma cell resistance and provides a treatment option with HDAC inhibition to break this resistance mechanism.

## Supplementary information

Statement of author contributions

Supplementary Tables and Figures

Reproducibility checklist

Supplementary material Western Blots

## Data Availability

The data needed to evaluate the conclusions in the paper are present in the paper. Additional data related to this paper may be requested from the corresponding author. The mass spectrometry proteomics data have been deposited to the ProteomeXchange Consortium via the PRIDE partner repository with the dataset identifier PXD028683.
