# Supplementary material for: Class I HDAC overexpression promotes temozolomide resistance in glioma cells by regulating RAD18 expression
Source: Cell Death Dis. 2022 Apr 1;13(4):293. doi: 10.1038/s41419-022-04751-7 (PMC8975953; doi:10.1038/s41419-022-04751-7)
Supplement: Supplementary file 1 — Statement of author contributions [file 41419_2022_4751_MOESM1_ESM.docx]

**Class I HDAC overexpression promotes temozolomide resistance in glioma cells by regulating RAD18 expression**

Daniela Hanisch, Andrea Krumm, Tamara Diehl, Carla M. Stork, Mario Dejung, Falk Butter, Ella Kim, Walburgis Brenner, Gerhard Fritz, Thomas G. Hofmann and Wynand P. Roos

**Authors’ contributions**

D.H., A.K., T.D., C.M.S., M.D., F.B., W.B. and W.P.R. performed the experiments. D.H., T.D., C.M.S., M.D., F.B., G.F., T.G.H. and W.P.R. analysed the data and revised the manuscript. D.H. and W.P.R. conceived and designed the research and wrote the manuscript. The authors read and approved the final manuscript.
