# Supplementary material for: Class I HDAC overexpression promotes temozolomide resistance in glioma cells by regulating RAD18 expression
Source: Cell Death Dis. 2022 Apr 1;13(4):293. doi: 10.1038/s41419-022-04751-7 (PMC8975953; doi:10.1038/s41419-022-04751-7)
Supplement: Supplementary file 2 — Supplementary Tables and Figures [file 41419_2022_4751_MOESM2_ESM.pdf]

Supplementary tables

Table S1: Primary Antibodies

Antigen	Dilution WB	Dilution IF	Catalog number	Source	RRID
RAD18 (D2B8)	1:1000	1:500	#9040	Cell Signalling	AB_2756446
ubPCNA(Lys164)(D5C7P)	1:750		#13439	Cell Signalling	AB_2798219
pp53 (Ser15)	1:1000		#9284	Cell Signalling	AB_331464
pCHK1 (Ser345)	1:1000		#2341	Cell Signalling	AB_330023
pCHK2 (Thr68)	1:1000		#2661	Cell Signalling	AB_331479
pATM(Ser1981) (10H11.E12)	1:1000	1:100	#4526	Cell Signalling	AB_2062663
HDAC1	1:1000		#5356	Cell Signalling	AB_10612242
HDAC2 (3F3)	1:1000		#5113	Cell Signalling	AB_10624871
HDAC3 (7G6C5)	1:1000		#3949	Cell Signalling	AB_2118371
TALIN (C45F1)	1:1000		#4021	Cell Signalling	AB_2204018
CD31 (Pecam1)	1:1000	1:500	#3528	Cell Signalling	AB_2160882
MGMT	1:1000		MAB16200	Millipore	AB_2281919
ACTIN (C4)	1:1000		sc-47778	Santa Cruz	AB_2714189
PCNA (PC10)	1:1000		sc-56	Santa Cruz	AB_628110
GFP	1:5000		sc-8334	Santa Cruz	AB_641123
HSP90 (F-8)	1:1000		sc-13119	Santa Cruz	AB_675659
p53 (DO-1)	1:1000		sc-126	Santa Cruz	AB_628082
vWF (EPSISR)	1:1000	1:250	ab154193	Abcam	
GFAP			Z0334	Dako	AB_10013382
NESTIN (196908)			MAB1259	R&D Systems	AB_2251304

Table S2: Secondary Antibodies

Antigen	Dilution WB	Dilution IF	Catalog number	Source	RRID
anti-mouse green	1:10000		926-32212	Li-COR Bioscience	AB_621847
anti-mouse-red	1:10000		925-68072	Li-COR Bioscience	AB_2814912
anti-rabbit green	1:10000		926–32213	Li-COR Bioscience	AB_621848
anti-rabbit Alexa Fluor 488		1:500	111-546-144	Jackson ImmunoResearch	AB_2338057
anti-mouse Cy3		1:500	115-165-146	Jackson ImmunoResearch	AB_2338690

Table S3: Plasmids used

**Plasmid**	**Gift from**	**Catalog number**	**Source**	**RRID**
pDRGFP	Maria Jasin	#26475	Addgene	Addgene_26475
pCβASceI	Maria Jasin	#26477	Addgene	Addgene_26477
hRAD18-EGFP	Satoshi Tateishi	#68824	Addgene	Addgene_68824

Table S4: Mass spectrometry conditions

Separation of peptides	Condition
25-cm capillary (New Objective)	Packed in-house with ReproSil-Pur C18-AQ 1.9-μm resin (Dr Maisch GmbH)
Peptides were separated using:	A 225 min gradient from 2% to 40% acetonitrile in 0.5% formic acid

Mass spectrometry	Condition
Spray voltage	Between 2.4 and 2.6 kV
The instrument was operated in:	Data-dependent mode (DDA) performing top 10 MS/MS per MS full scan
Isotope patterns:	Unassigned and charge state 1 were excluded
MS scans:	70000 resolution
MS/MS scans:	17500 resolution

Data analysis	Condition
MaxQuant (version 1.5.2.8)	Default settings
LFQ was activated	LFQ min. ratio count of 2
Carbamidomethyl	Fixed modification
Acetylation and oxidation	Variable modification
Quantification	Unique peptides only
Match between runs	Activated with 0.7 minutes matching time window and 20 minutes alignment time window
Data base	Homo sapiens Ensembl protein database (release 95)
MaxQuant results filtering:	Removed all reverse database hits and known contaminants and proteins only identified by site modification
Additional filtering:	All protein groups detected with less than 2 peptides (minimum 1 unique) and protein groups not quantified in at least 2 replicates in one condition were removed
Missing LFQ values:	Imputed with a beta distribution in a range between 0.2 and 2.5 percentile of measured sample in each replicates separately
Stats:	Fold change was calculated by the mean average of the replicates. P-values were calculated using Welch two-sample t-test.
Gene ontology analysis:	R packages GOstats and GSEABase with a p-value cut-off of 0.01 for the hypergeometrical test function.

Table S5: Microarray datasets used for GENT2 analysis

GSE68848	GSE61374	GSE74195	GSE15824	GSE30563
GSE35493	GSE4536	GSE43289	GSE62802	GSE9438
GSE68015	GSE4290	GSE66354	GSE19578	GSE26576
GSE43378	GSE73038	GSE64415	GSE36782	GSE49822
GSE29796	GSE55609	GSE43388	GSE49243	GSE45921
GSE19404	GSE67851	GSE31545	GSE67066	GSE51413
GSE60184	GSE37418			

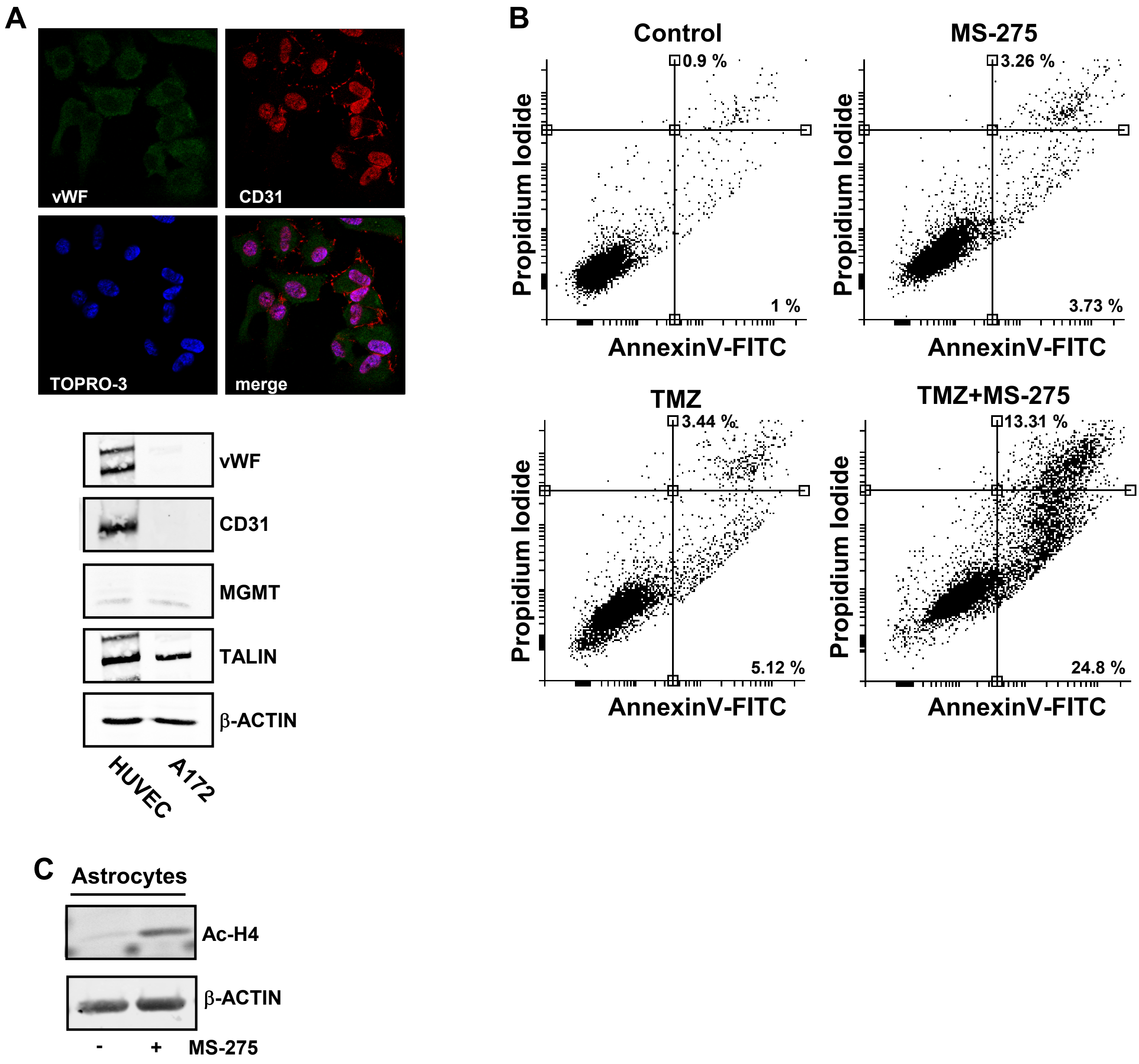

A
vWF
CD31
TOPRO-3
merge
vWF
CD31
MGMT
TALIN
β-ACTIN
HUVEC
A172
B
Control
MS-275
TMZ
TMZ+MS-275
Propidium Iodide
AnnexinV-FITC
0.9 %
1 %
3.26 %
3.73 %
3.44 %
5.12 %
13.31 %
24.8 %
C
Astrocytes
Ac-H4
β-ACTIN
-
+
MS-275

Figure S1

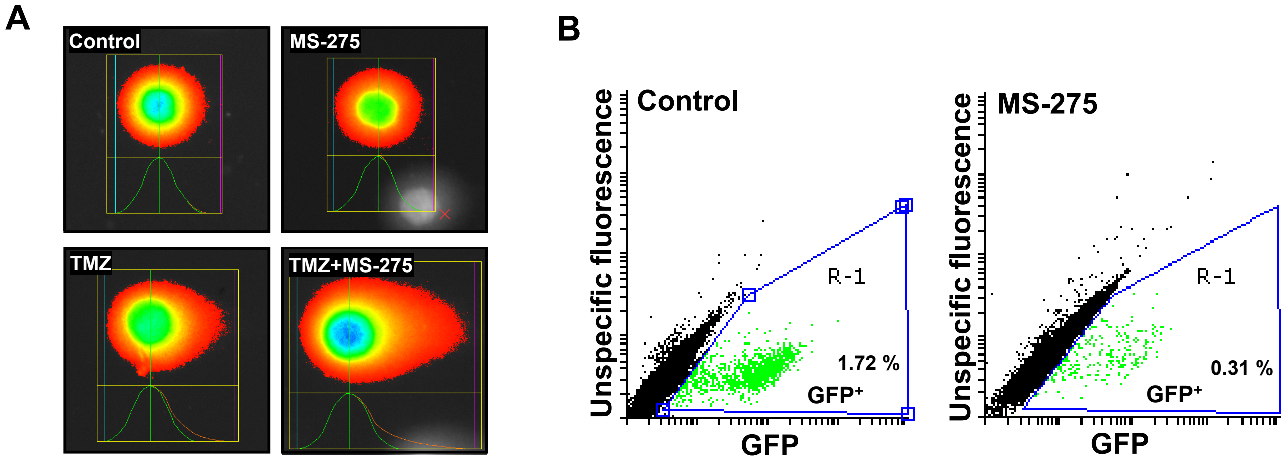
A
Control
MS-275
TMZ
TMZ+MS-275
B
Control
MS-275
Unspecific fluorescence
R-1
1.72 %
0.31 %
GFP+
GFP

Figure S2

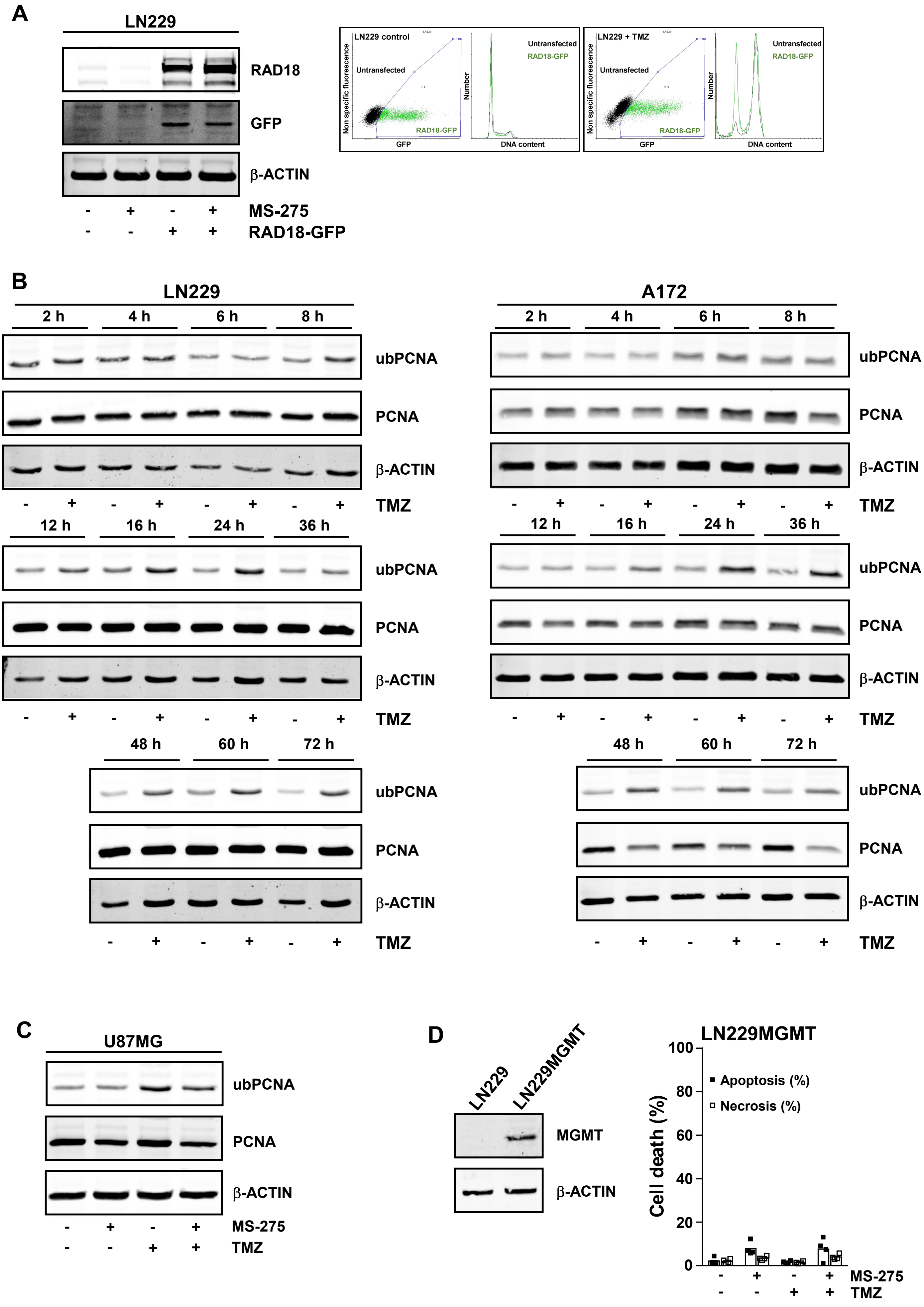
A
LN229
RAD18
GFP
β-ACTIN
MS-275
RAD18-GFP
LN229 control
Untransfected
RAD18-GFP
Non specific fluorescence
GFP
Number
DNA content
LN229 + TMZ
B
LN229
A172
2 h
4 h
6 h
8 h
12 h
16 h
24 h
36 h
48 h
60 h
72 h
ubPCNA
PCNA
TMZ
C
U87MG
D
LN229
LN229MGMT
MGMT
Apoptosis (%)
Necrosis (%)
Cell death (%)
0
20
40
60
80
100

Figure S3

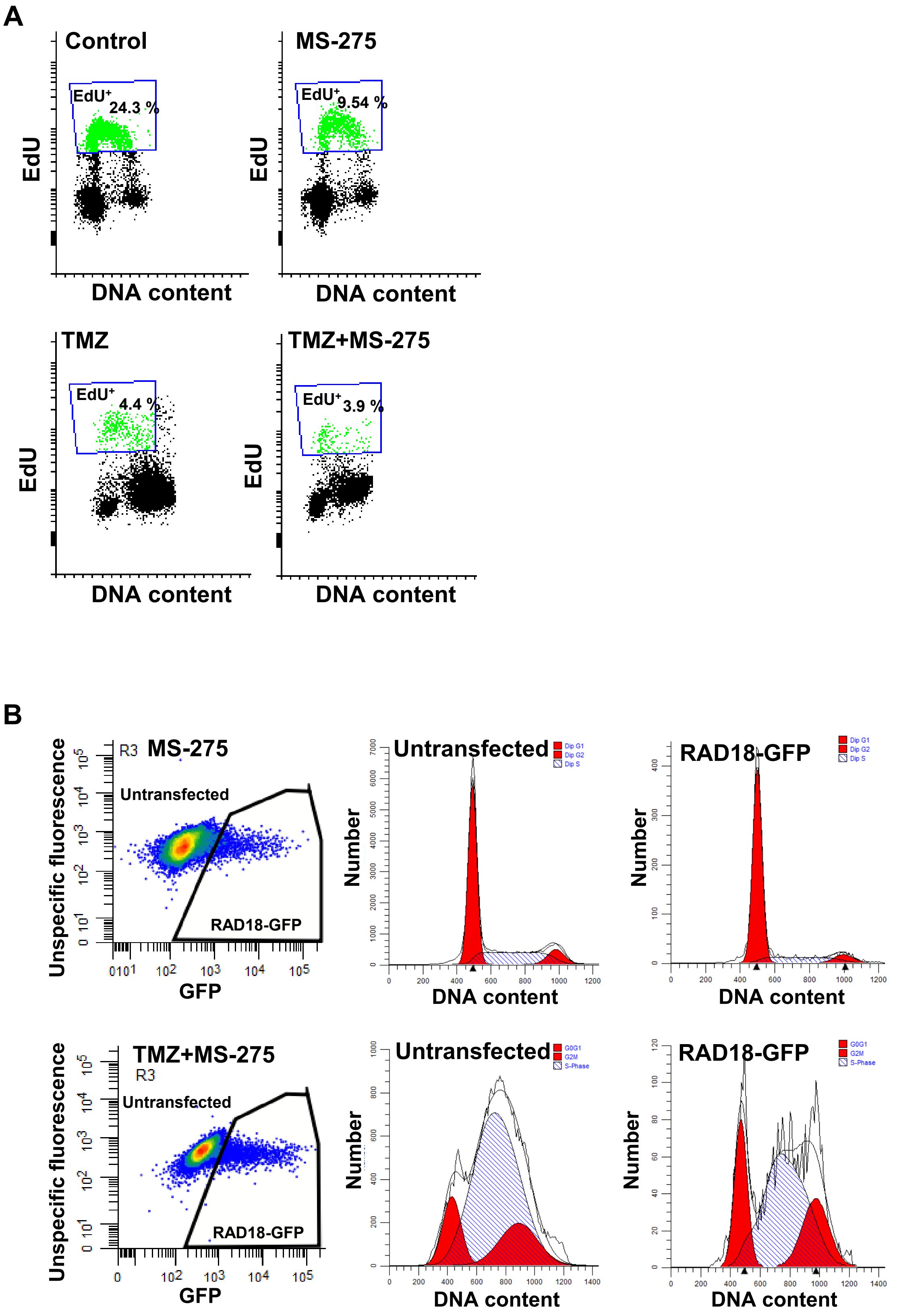
A
Control
MS-275
TMZ
TMZ+MS-275
EdU
EdU+
24.3 %
9.54 %
4.4 %
3.9 %
DNA content
B
R3
MS-275
TMZ+MS-275
Untransfected
RAD18-GFP
Unspecific fluorescence
GFP
Number
DNA content
Dip G1
Dip G2
Dip S
G0G1
G2M
S-Phase

Figure S4

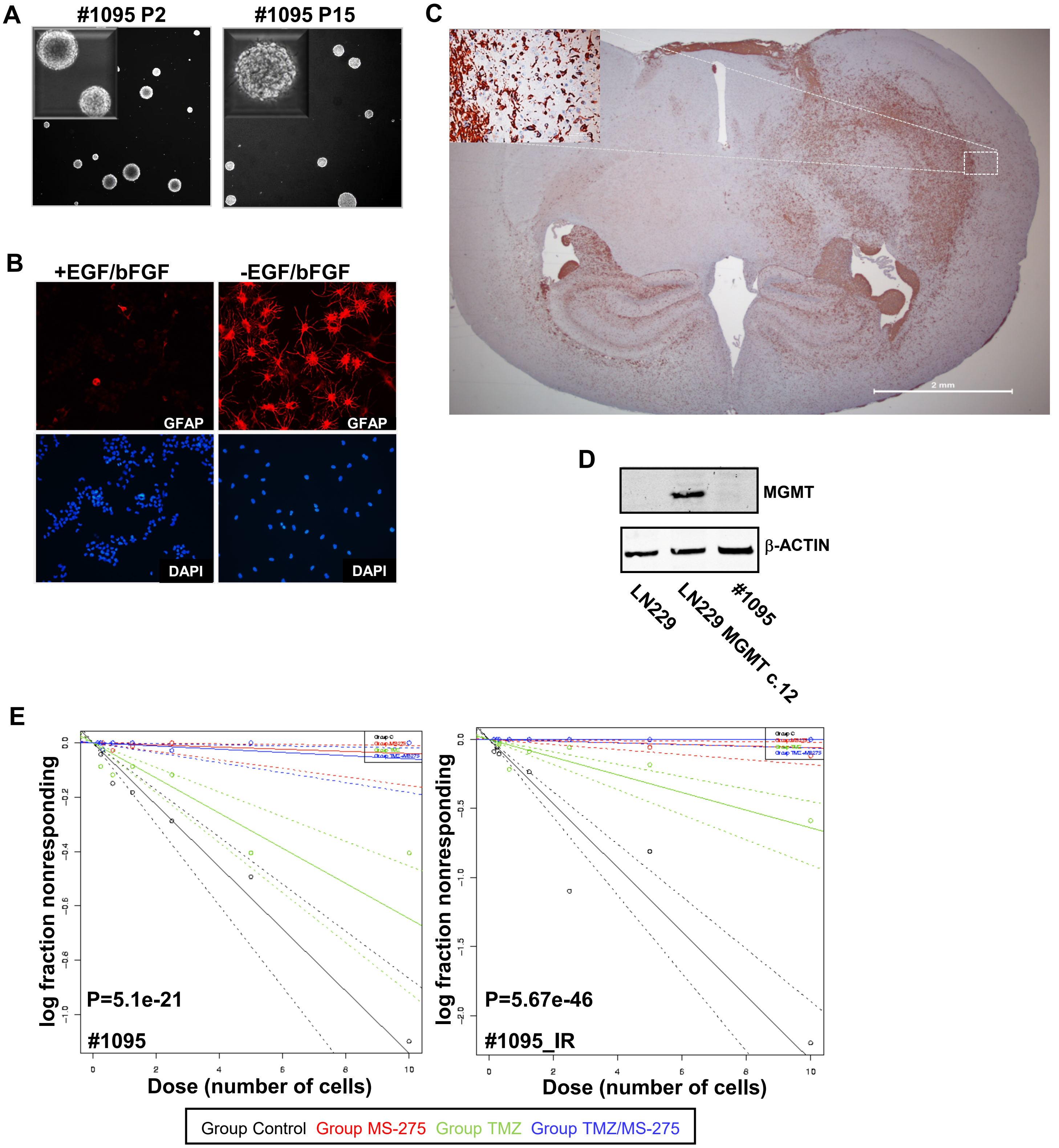

F

#1095

Pairwise test for differences in stem cell frequency

Compared Groups	Chisq	Pr(>Chisq)
Control – MS-275	64.2	1.12e-15
Control - TMZ	6.61	0.0101
Control – TMZ/MS-275	59.3	1.36e-14
MS-275 - TMZ	32.9	9.67e-09
MS-275 – TMZ/MS-275	0.203	0.653
TMZ – TMZ/MS-275	29	7.24e-08

#1095_IR

Pairwise test for differences in stem cell frequency

Compared Groups	Chisq	Pr(>Chisq)
Control – MS-275	134	6.93e-31
Control - TMZ	45.5	5.38e-12
Control – TMZ/MS-275	153	4.5e-35
MS-275 - TMZ	28.4	9.87e-08
MS-275 – TMZ/MS-275	4.04	0.0445
TMZ – TMZ/MS-275	43.4	4.52e-11

Figure S5

Supplementary Figure Legends

Figure S1

A, Verification of HUVEC lineage by determining the expression of the von Willebrand factor (vWF) and CD31. **Top**, Micrographs showing the results of immunofluorescent staining against vWF and CD31. Nuclei were stained with TO-PRO-3. **Bottom**, Western blot analysis determining the expression of vWF, CD31 and MGMT in HUVECs compared to glioma cells (A172). ß-ACTIN and TALIN served as loading controls. **B**, representative flow cytometry dot plots showing Annexin V-FITC/PI double-stained cells 120 h after the exposure to MS-275 (1.5 µM), TMZ (50 µM) and TMZ/MS-275. Percentages of apoptotic and necrotic cells are indicated. **C**, Western blot analysis of human, primary astrocytes determining the influence of class I HDAC inhibition by MS-275 (1.5 µM for 72 h) on acetylated-H4. ß-ACTIN served as a loading control.

Figure S2

A, Micrographs showing the results of the neutral comet assay in LN229 cells exposed to MS-275 (1.5 µM), TMZ (50 µM) and TMZ/MS-275 for 72 h. **B**, HR activity after inducing a DSB by transient transfection with a SceI expressing plasmid and MS-275 exposure. Representative flow cytometry dot plots showing GFP and autofluorescence following MS-275 exposure in LN229 DRGFP c.46. Percentages of GFP^+ cells are indicated.

Figure S3

A, Expression of the RAD18-EGFP plasmid in LN229 cells. **Top**, Western blot analysis showing the expression of the RAD18-EGFP plasmid in LN229 cells. ß-ACTIN served as a loading control. **Bottom**, representative flow cytometry dot plots showing GFP and autofluorescence of LN229 cells exposed to TMZ (50 µM). Comparative overlay of histograms showing the Sub-G_1 fraction of untransfected and transfected cells within the same sample. **B**, Western blot analysis showing the mono-ubiquitination of PCNA induced by TMZ (50 µM) after indicated time points in LN229 and A172 cells. ß-ACTIN served as a loading control. **C**, HDACi prevents TMZ-induced mono-ubiquitination of PCNA as determined by Western blot analysis. Glioma cells (LN229, A172) were exposed to indicated drugs. ß-ACTIN served as a loading control. **D**, HDACi sensitizes glioma cells towards the O^6MeG lesion induced by TMZ. Following MGMT reconstitution, assessed by Western Blot analyses (Top), the cell death response of LN229 MGMT cells upon indicated drug exposure was determined (Bottom).

Figure S4

A, representative flow cytometry dot plots showing incorporated EdU and DNA content (DAPI) in LN229 cells exposed to indicated drugs for 72 h. Percentage of EdU^+ cells is indicated. **B**, representative flow cytometry dot plots showing RAD18-EGFP and autofluorescence of LN229 cells exposed to MS-275 and TMZ/MS-275 and comparative histograms showing the cell cycle distribution of untransfected and transfected cells within the same sample. Cells were exposed to MS-275 (1.5 µM), TMZ (50 µM) and TMZ/MS-275. Cell cycle distribution was determined by Sub-G_1 analysis and flow cytometry.

Figure S5

Characterization of patient-derived glioma initiating cells #1095 and the effect of MS-275 and TMZ on #1095 and its radiation-resistant counterpart #1095_IR.

A, Micrographs showing the maintenance of self-renewal potential in the presence of self-renewal promoting factors bFGF and EGF under serum-free culture conditions. Magnification 1.6x. Enlarged images show glioma spheres formed at clonal densities (1 cell/ml) at passage 2 or 15. **B**, Micrographs showing the results of immunofluorescent staining for GFAP in #1095 cells cultured in presence and absence of bFGF/EGF. The differentiation capacity of #1095 cells is observed upon bFGF/EGF withdrawal. The astrocytic lineage marker GFAP (red) served as a marker for differentiation. Nuclei were stained with DAPI. **C**, Representative xenograft grown from #1095 cells implanted into the brain of nude mice showing the tumor-initiating capacity. Immunohistochemical staining for human NESTIN (brown staining). Magnification 1.6x. Enlarged micrograph showing tumor invasion into the brain parenchyma, a characteristic phenotype of GBMs. **D**, Western blot analysis determining the MGMT expression of #1095 cells. LN229 and LN229 MGMT c.12 served as a negative and positive control respectively for MGMT expression. ß-ACTIN served as a loading control. **E**, Panels show the results of self-renewal potential of #1095 and #1095_IR cells determined by ELDA. Panels show the results from three independent experiments. Cells were exposed to MS-275 (1.5 µM), TMZ (50 µM) and TMZ/MS-275. The p-values indicate the significant difference in stem cell frequency between groups and result from Chi-Square test performed by ELDA. **F**, Pairwise Chi-Square test for differences in stem cell frequencies of indicated groups performed by ELDA and the resulting Chisq-and p-values.
