# Supplementary figures and images for: Class I HDAC overexpression promotes temozolomide resistance in glioma cells by regulating RAD18 expression
Source: Cell Death Dis. 2022 Apr 1;13(4):293. doi: 10.1038/s41419-022-04751-7 (PMC8975953; doi:10.1038/s41419-022-04751-7)

**B**

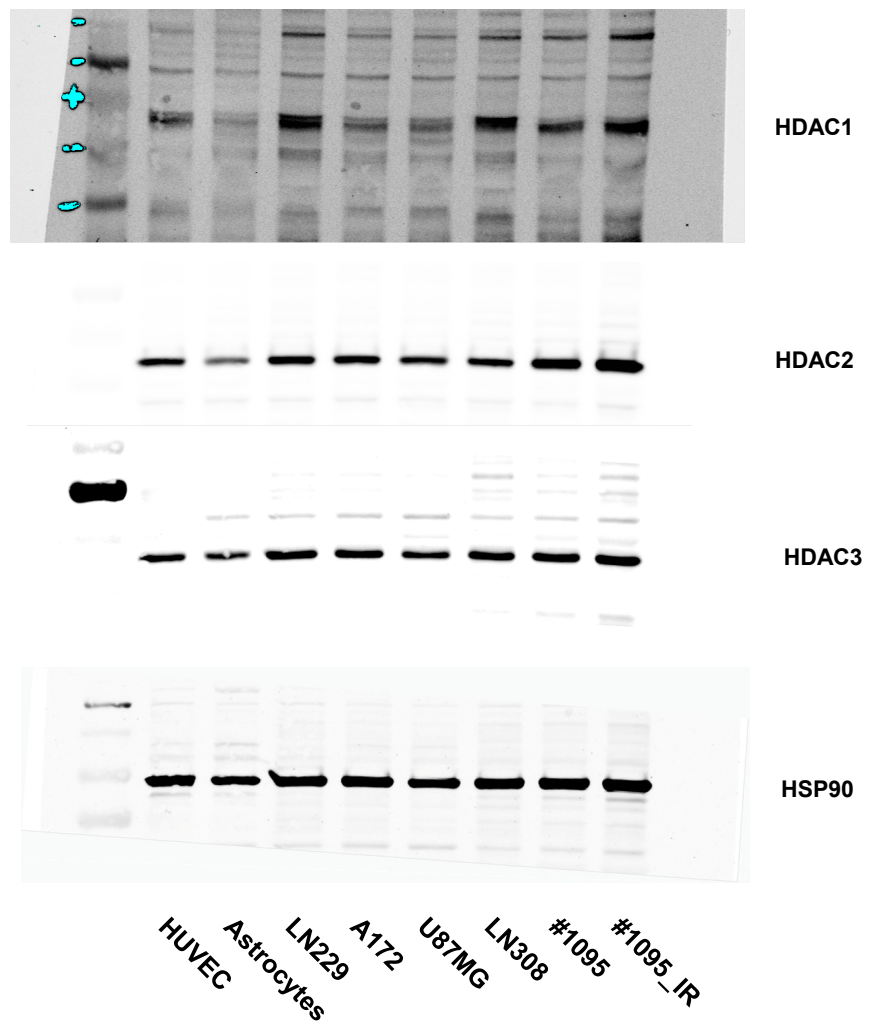

Figure 1

C

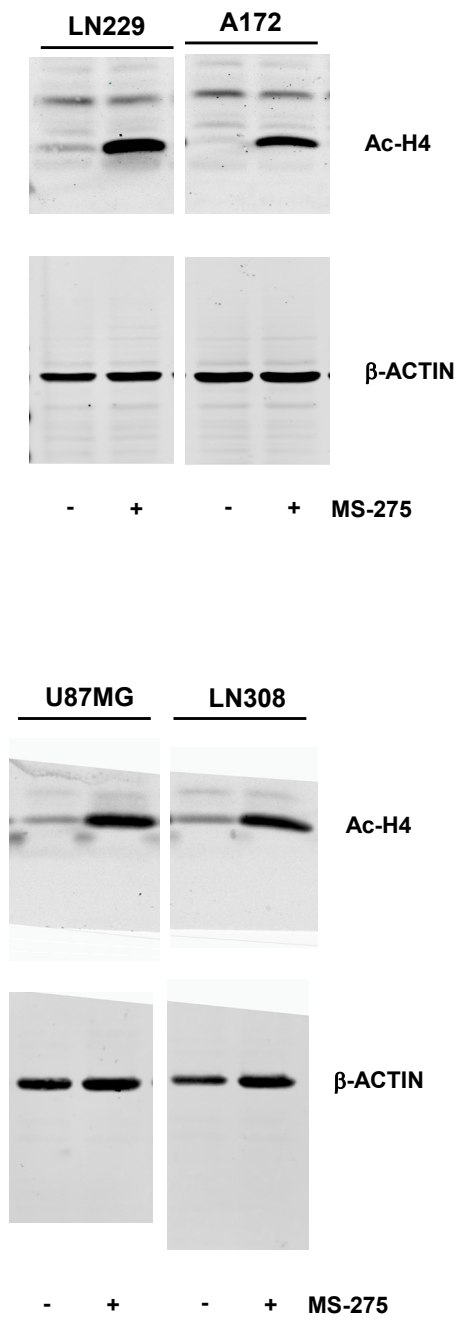

Figure 1

**A**

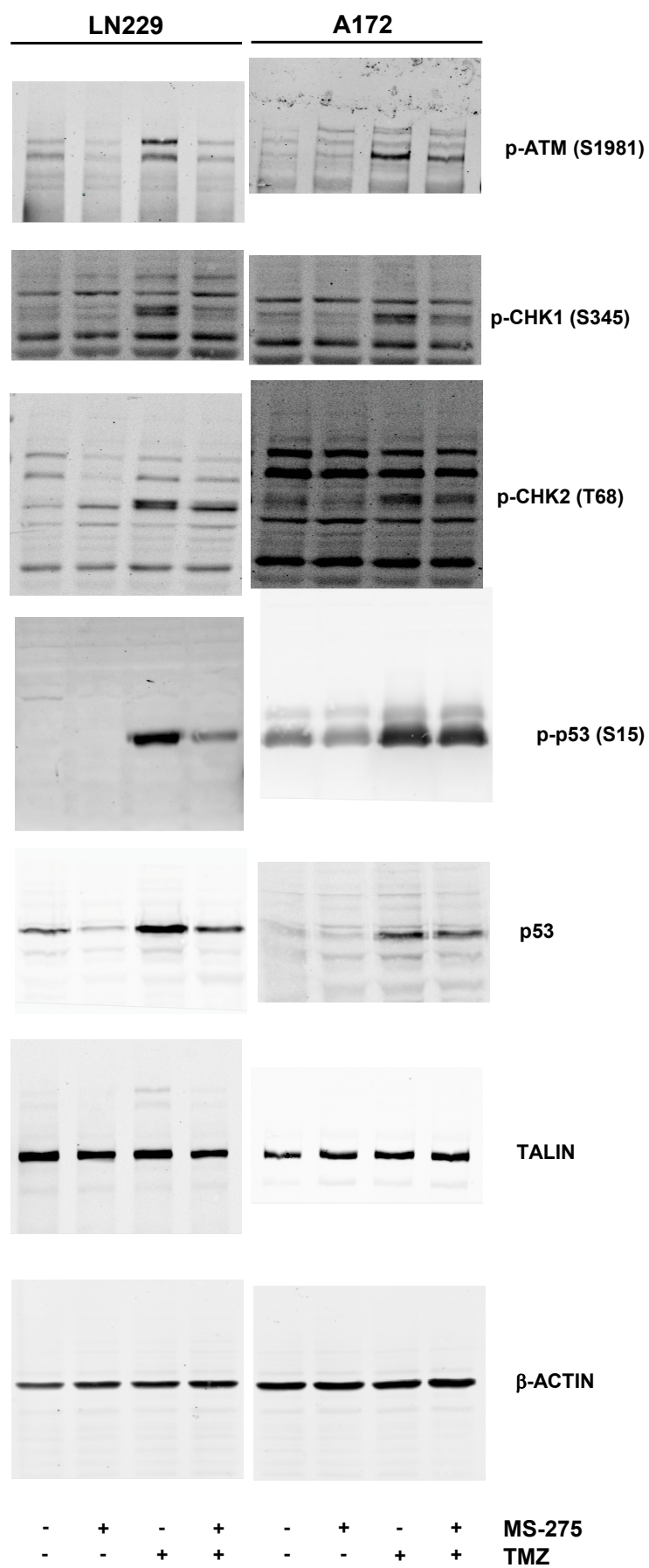

Figure 2

**B**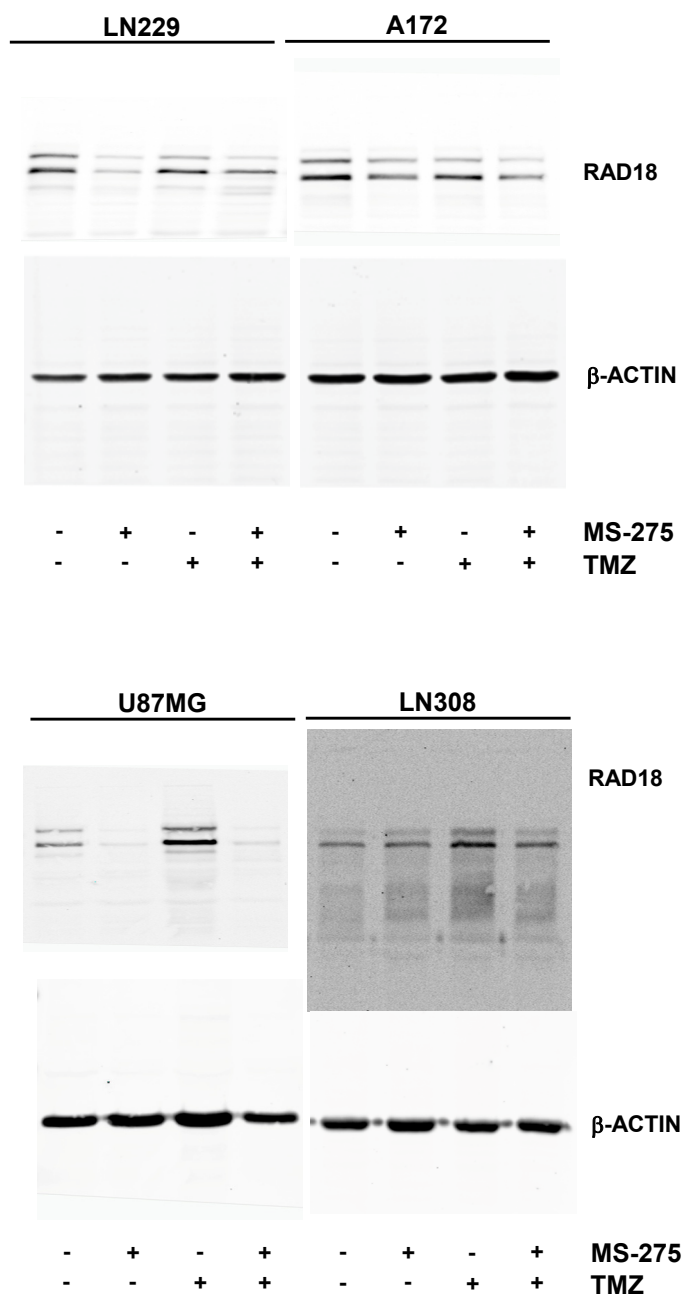

Figure 3

C

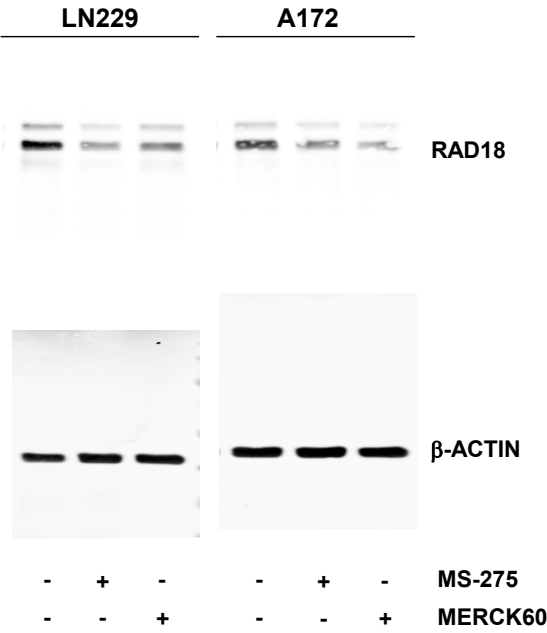

Figure 3

D

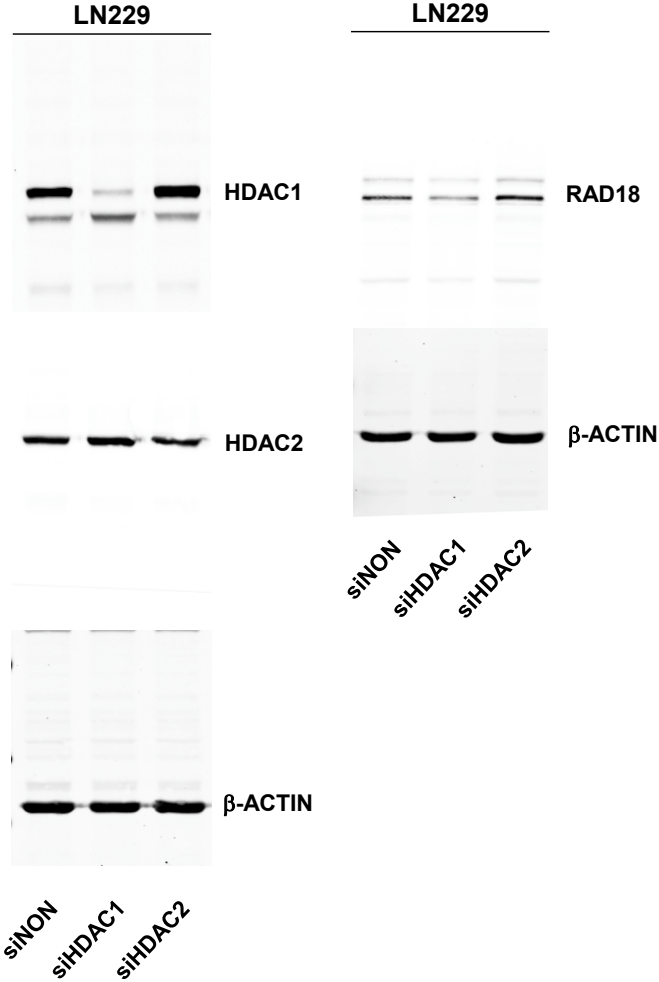

Figure 3

**B**

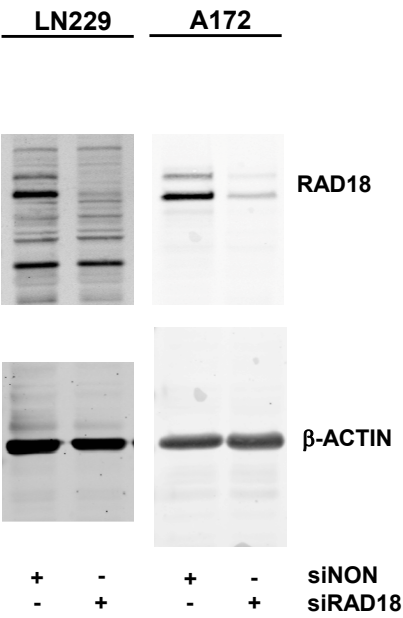

Figure 4

D

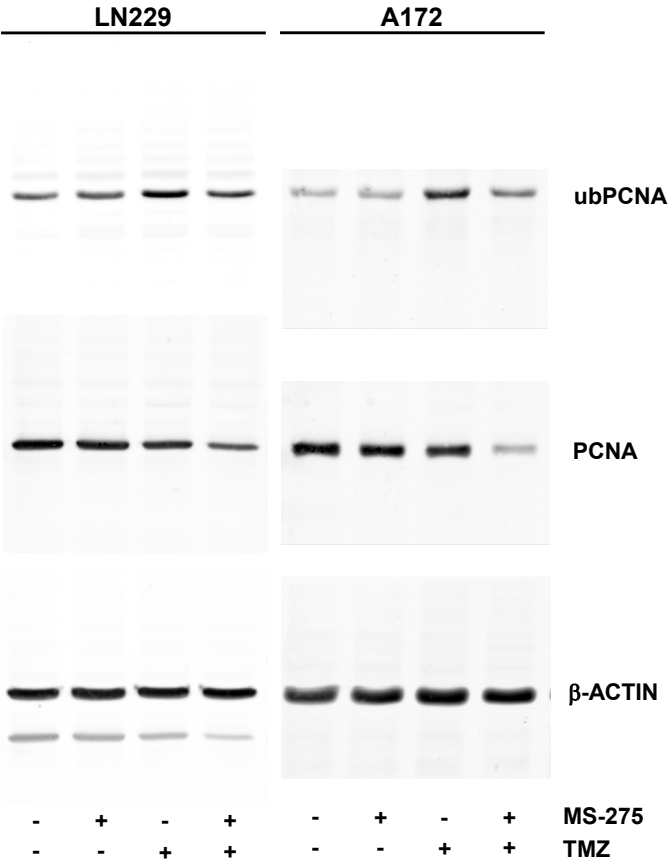

Figure 4

E

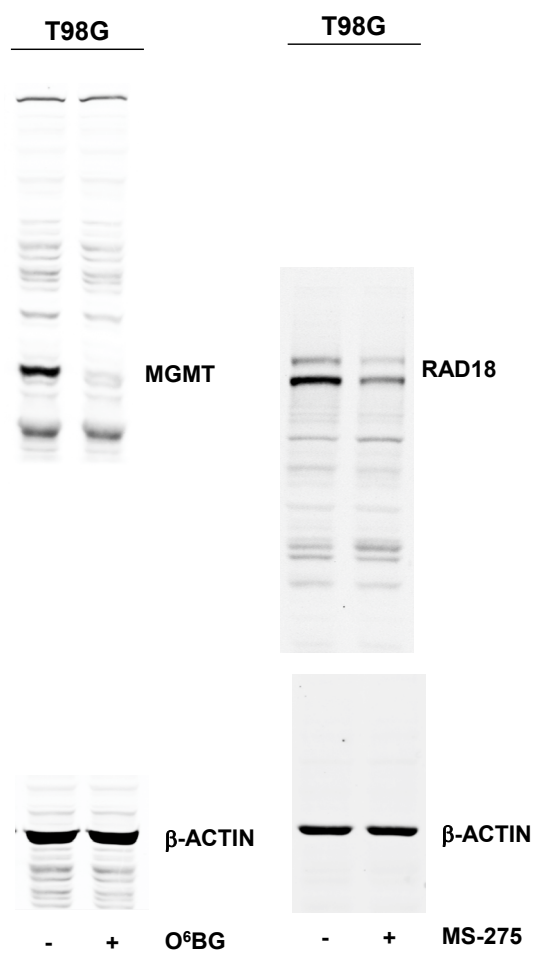

Figure 4

**A**

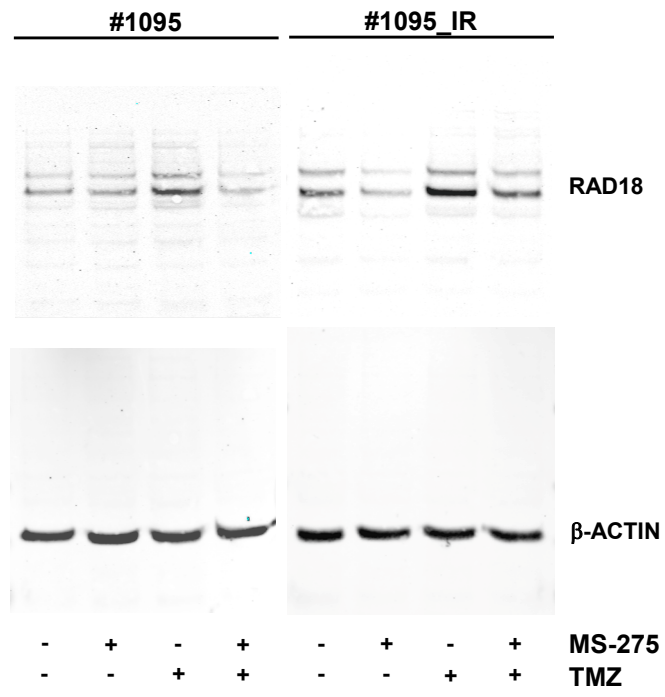

Figure 6

Supplement: Supplementary file 4 — Supplementary material Western Blots [file 41419_2022_4751_MOESM4_ESM.pdf]
